# Considering best practices in color palettes for molecular visualizations

**DOI:** 10.1515/jib-2022-0016

**Published:** 2022-06-22

**Authors:** Laura Garrison, Stefan Bruckner

**Affiliations:** Department of Informatics, University of Bergen, Bergen, Norway

**Keywords:** biomedical illustration, color palette, design, molecular visualization

## Abstract

Biomedical illustration and visualization techniques provide a window into complex molecular worlds that are difficult to capture through experimental means alone. Biomedical illustrators frequently employ color to help tell a molecular story, e.g., to identify key molecules in a signaling pathway. Currently, color use for molecules is largely arbitrary and often chosen based on the client, cultural factors, or personal taste. The study of molecular dynamics is relatively young, and some stakeholders argue that color use guidelines would throttle the growth of the field. Instead, content authors have ample creative freedom to choose an aesthetic that, e.g., supports the story they want to tell. However, such creative freedom comes at a price. The color design process is challenging, particularly for those without a background in color theory. The result is a semantically inconsistent color space that reduces the interpretability and effectiveness of molecular visualizations as a whole. Our contribution in this paper is threefold. We first discuss some of the factors that contribute to this array of color palettes. Second, we provide a brief sampling of color palettes used in both industry and research sectors. Lastly, we suggest considerations for developing best practices around color palettes applied to molecular visualization.

## 1 Introduction

Suppose you have landed a project for the visualization of the mechanism of action of a novel cancer treatment drug that is about to come to market. One of the key elements of the brief is showing how the drug acts at a molecular level. The visualization you produce must be accurate, as well as beautiful, informative, and memorable: your client wants hospital administrators, doctors, and patients to be interested in, and to opt in to, this drug. In telling this story, color palette will be one of the main and most challenging creative decisions you will have to make in the production process. The brief may include a suggested color palette that aligns with the drug’s branding, or you may be free to choose colors that you feel are most appropriate to tell the story that you want to tell. If you choose poorly, you risk a visualization that is unappealing, ineffective, or incomprehensible. If you are a biomedical illustrator, this is a common scenario that you are faced with. As a researcher who works with molecules, aspects of this scenario may also be quite familiar. Color selection can be overwhelming, particularly for novices, and outside of standard best practices for color there are no general guidelines in place for the coloring of molecules. This paper identifies some of the rationale for the broad use of color in molecular visualizations, provides a set of contemporary color palette examples, and discusses considerations towards best practices to lead to more interpretable, accurate, and consistent molecular visualizations without compromising on aesthetics or overly limiting creative freedom.

**Figure 1:**
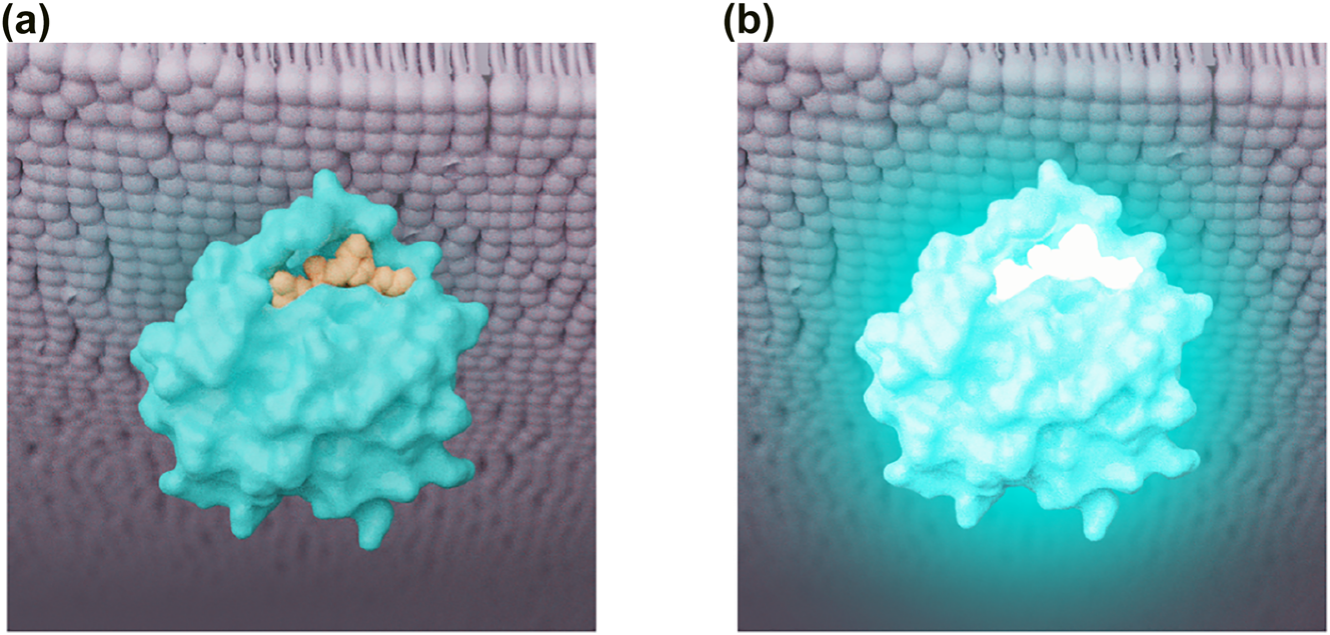
Two color choices and effects to illustrate a molecular reaction between a ligand and its protein receptor. (a) Orange ligand with similar luminance and saturation to its receptor. (b) Ligand and receptor with higher luminance and saturation, with additional highly saturated glow effect.

**Figure 2:**
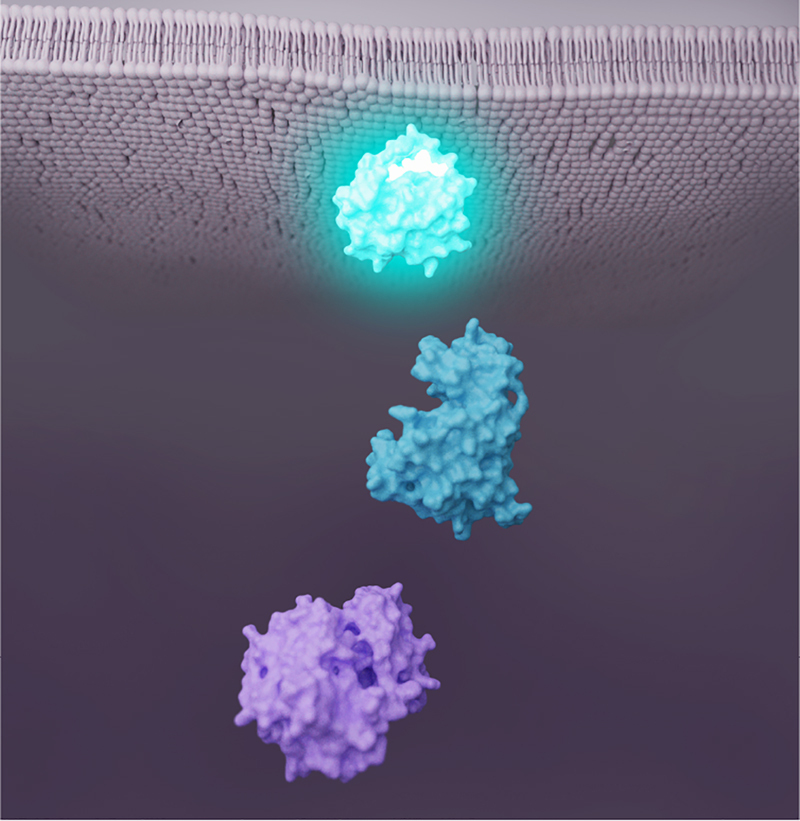
Simple biomedical illustration depicting key molecules in a pathway.

Visualizations can tell stories that aid in exploration, analysis, and communication of complex molecular phenomena to a range of audiences and user types [1–3]. For this paper, we generalize to three story features in a molecular visualization where color is an important consideration:

1. **Focus + context molecules.** Molecular visualizations are often structured in a visual hierarchy such that *focus* molecules are shown prominently and in full detail. *Context* molecules or structures are de-emphasized and provide an overview of and add visual interest to the scene.
  Both Figure 1a and b use color to help develop this hierarchy. Color increases the prominence of the focus molecules (ligand and receptor) and allows the context molecules (lipid bilayer of the cell membrane) to recede into the background while still providing locational context for the scene.
2. **Molecular reaction(s).** Molecules can interact in reactions that fundamentally change their properties, synthesize new molecules, or destroy molecules. A specific and commonly–visualized scenario is *ligand binding*, where a *ligand* is defined as any substance that forms a complex with another molecule to serve a biological purpose [4]. The specific region of the molecule that the ligand binds to is known as a *binding site.* This step initiates (or blocks) a series of reactions that contribute to pathways integral to the life cycle and behavior of a cell, with natural implications in drug development and protein engineering research. Figure 1 shows two different color approaches for this event that experiment with saturation and luminance to draw attention and semantically connect to the concept of “binding and activation.”
3. **Molecular pathway.** A sequence of molecular reactions, often which are initiated by a ligand binding event, describe a molecular pathway. Understanding molecular pathways and their functions is critical to understanding the functioning of higher-order structures such as cells, tissues, organs, organ systems, and even the entire body. An example of this is shown in Figure 2 with three key molecules in a given intracellular pathway. Color helps provide functional semantics to the visualization: similar colors show that the three molecules are connected, and a color progression indicates the order of the molecules in the pathway.

**Figure 3:**
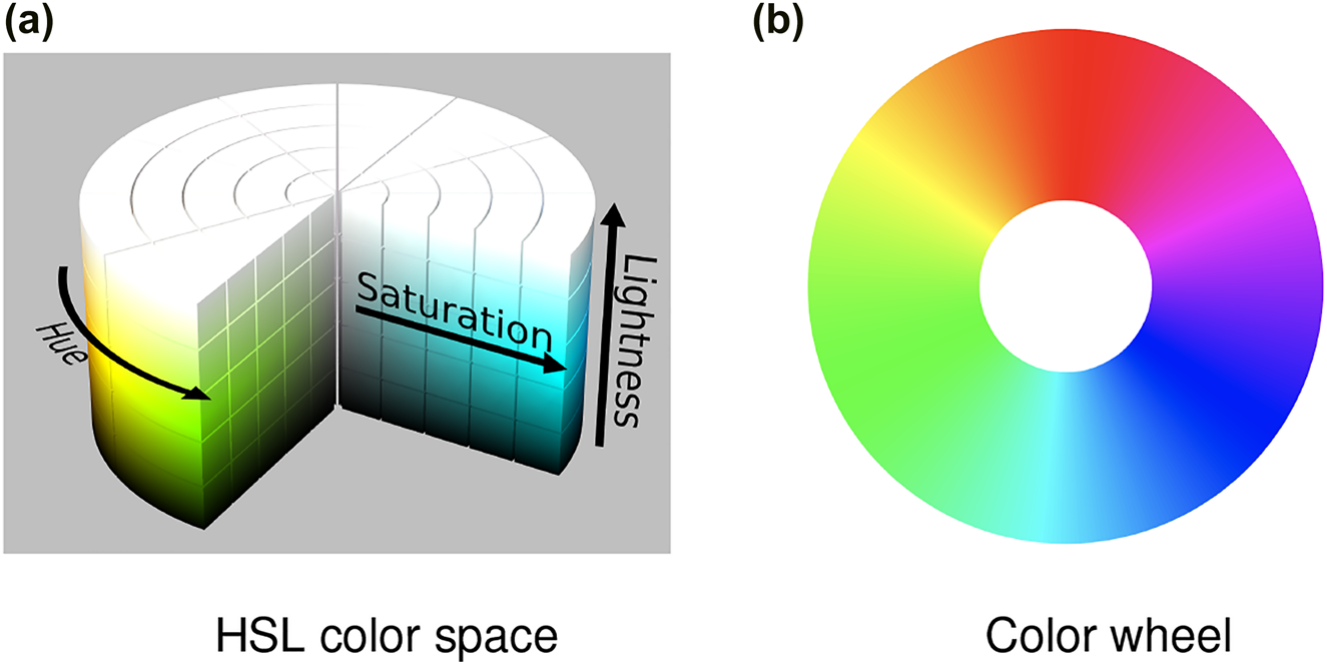
Color. (a) Source: https://en.wikipedia.org/wiki/HSL_and_HSV. (b) Adapted from: https://commons.wikimedia.org/wiki/File:RGB_color_wheel_360.svg.

Color plays a vital role in conveying each of these story features. While it can be represented in several formats, such as RGB (red, green, blue), CMYK (cyan, magenta, yellow, key: black), or HSL (hue, saturation, lightness) [5], for the purposes of this paper it is most intuitive and useful to think of color in the HSL color space. This encompasses the three color properties depicted on the 3D cylinder shown in Figure 3a. Hue specifies a base color, e.g., cyan, that is localized by angle around the color wheel illustrated in Figure 3b. Saturation defines the purity of a hue. Values span the inner to outer perimeter of the cylinder, from no saturation (grey) to full saturation, e.g., pure cyan. Lightness specifies color brightness, ranging from the bottom (black) to the top of the cylinder (white). Mixing black into a color produces a shade, while blending with white produces a tint.

**Figure 4:**
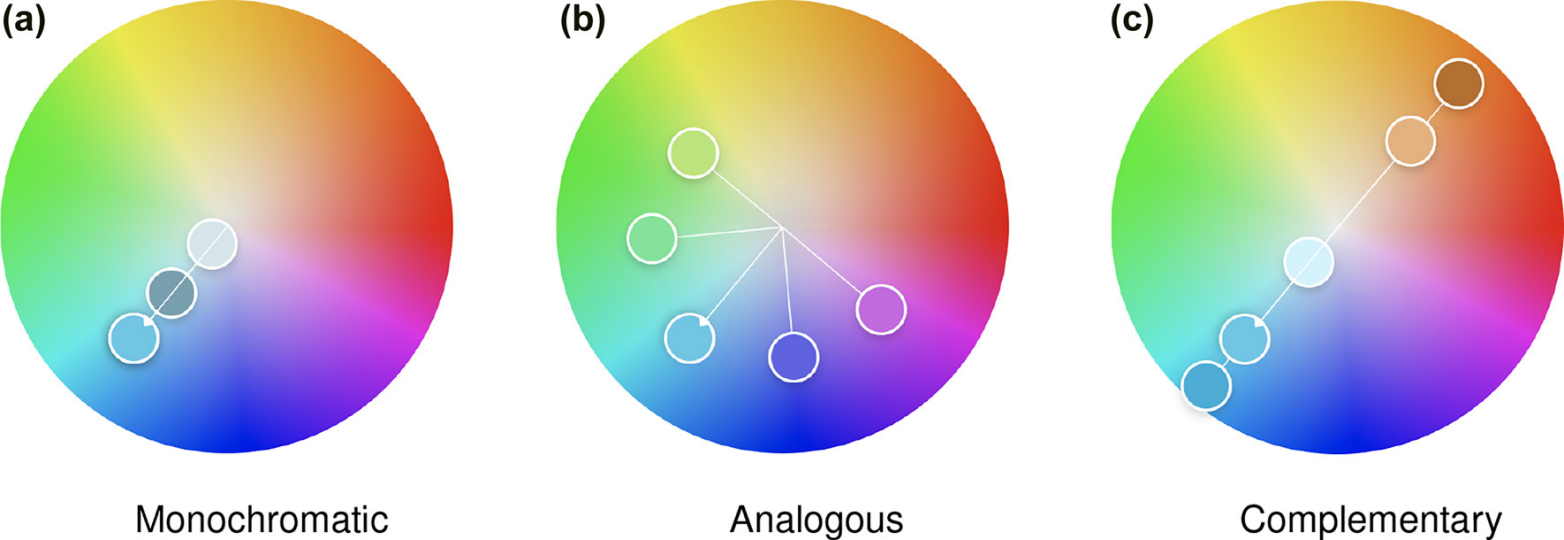
Three common color harmony rules with base color blue. Created in Adobe Color: https://color.adobe.com/create/color-wheel.

A color palette is the combination of colors used to design a visualization. A number of color harmony rules aid in creating visually pleasing palettes. Derived from Itten’s seven models of color contrast [6], harmony rules may be *monochromatic*, *analogous*, or *complementary*, among others. *Monochromatic* palettes are formed from tints and shades of a single color, as in Figure 4a. *Analogous* palettes comprise colors that are adjacent on the color wheel, as in Figure 4b. This type of palette is employed in Figure 2 to indicate that the molecules are part of the same pathway, and are therefore functionally connected. *Complementary* palettes are comprised of colors that are opposite each other on the color wheel, as in Figure 4c. Colors from these palettes can be used to draw attention to a particular element, to guide the eye through a narrative, or to establish a visual hierarchy of focus + context elements in a molecular visualization.

While the artist often has creative license to choose their palette, other factors come into play. Clients from different sectors have different aims. A pharmaceutical company has different requirements than an educational or research institution. The intended audience may have certain cultural sensitivities, visual or cognitive disabilities, or other factors to consider. While on an individual basis these requirements can provide guard rails that limit the color design space, color selection remains broad and inconsistent overall. This leads to diluted semantic meaning of molecular structures, which can impact a visualization’s interpretability and effectiveness on a larger scale. For example, if COVID-19 spike proteins are colored blue, rather than the red used in the well-known version produced by Alissa Eckert and Dan Higgins for the CDC,1
https://phil.cdc.gov/Details.aspx?pid=23311. can everyone still recognize it as the COVID-19 virus? What are the consequences if they can’t?

Many education and research-oriented applications use the CPK coloring convention for atoms [7] when atomic-scale resolution is key to the visualization. At the cellular scale there are established colors for certain cell types, where the red blood cell is perhaps the most obvious example. This is always red, unless there is an express reason to show it otherwise, e.g., deoxygenated cells. Immune cells are often shown in cool colors that echo the soothing blue color often seen in the medical field. Molecules can be similarly classified, to some extent, into related groups according to structure or function. With a standard in place for semantically coloring atoms, and an informal semantic coloring practice for coloring cells, why not have something in place for molecules? Limited works in visualization have addressed color treatment in molecular visualizations. These consider the use of illumination models to cue features on molecular surfaces [8, 9] and coloring of multiscale molecular visualizations [10, 11]. While the application of high luminance colors to focus objects is a consistent recommendation of these works, color assignment on the whole is largely arbitrary and lacks consistent semantic meaning. Such works, alongside the broad community of professionals who craft them, can form the foundation for a set of best practices. This would enable easier creation of molecular visualizations that are more interpretable and effective, as well as aesthetically-pleasing.

## 2 Related work

In this section we briefly discuss works that explore color associations, as well as the use of color in molecular visualizations within the areas of biomedical illustration and scientific visualization.

Color can elicit different emotional and psychological reactions and associations [12, 13]. According to Itten, in general, ‘all tints (light colors) represent the brighter and better aspects of life, whereas shades (dark colors) symbolize the dark and negative forces [6].’ However, different cultures have often different affective interpretations of color. This is well-summarized in the visualization “Colours in Culture” by David McCandless and AlwaysWithHonor.com.2
https://informationisbeautiful.net/visualizations/colours-in-cultures/. In this graphic, we see the color black associating with, e.g., *intelligence* for Asian cultures and *style* for Japanese and Hindu cultures. Even in the same culture, a color can take on different meaning in different contexts, suggesting a more subjective and nuanced interpretation of color. Considering black again for Native American cultures in the “Colours in Culture” graphic, we see it associates with both *balance* and *death*. As a Western culture example, Wexner’s [14] study of color–mood associations with 94 psychology students at Purdue University found that participants strongly associated the color black to *despondent, dejected, unhappy, melancholy* as well as *powerful, strong, masterful.* Adams & Osgood [12] conducted a ground-breaking study on the affective meanings of color across 23 different cultural groups using bipolar adjectives. These adjectives were grouped into three factors: *Evaluation (E)*, *Potency (P)*, and *Activity (A)*. Examples for each factor include: good ↔ bad for *E*, strong ↔ weak for *P*, and active ↔ passive for *A*. They explored the perception of color in general, as well as seven distinct colors: white, grey, black, red, yellow, green, and blue. Among their findings, blue, white, and green were associated across nearly all 23 cultures as *good*, while black and grey more typically associated with *bad*. Black and grey furthermore were associated with *passive*, implying a degree of subjectivity in assessing the mood of these colors. Red is considered a strongly *active* color, although cultures disagree on its *evaluation*. Yellow exposes similar cultural disagreements on its *evaluation*. Filmmakers frequently take advantage of such color–mood associations to define the tone or mood of a film. Wei et al. [15] analyze the consistency of this on a global (entire film) and local (short shot sequences) scale. In this study, the authors collected and classified multidimensional feature vectors, including movie pace, movie dynamics, and dominant color ratio, to determine the mood of a film. Using the color-mood associations developed by Mahnke [13], an American psychologist, their approach found approximately 80% accuracy for mood/film genre classification according to color.

Color affect is also well-studied in visualization, with studies such as Bartram et al.’s work on the strength of associations between certain color palettes and affective response [16]. For example, *calm* often associates with cool colors with high lightness and low saturation. This strong association was found again in a related study by Kulahciogu & de Melo [17] investigating affective word clouds. In contrast, *playful* or *exciting* do not exhibit such distinct color palette associations, and Bartram et al. [16] note the need for more nuanced analyses of color harmony patterns. Color meaning can also be highly individual, particularly when associated with concepts or lesser-known objects [18]. In their crowd-sourced study of color palette selection for visualizations, Ahmad et al. [19] demonstrate this in quotes from two users who differ on the meaning of blue, red, and white in terms of *agree*, *disagree*, and *neutral*. However, color can also have strong semantic associations that postively impact performance. Lin et al. [20] found a faster response time for comparison–related tasks when data are assigned semantically meaningful colors for fruits (e.g., yellow ↔ banana), drinks, vegetables, and brands. For concepts or objects which are often lesser known and lack such strong semantic associations for the public, Schloss et al. [21] have shown that, with sufficient context, audiences can still infer meanings of colors. This holds promise for molecules, which are often unfamiliar to the public. Although there remain myriad reasons for subjective and variable interpretations of color semantics, e.g., culture, color blindness, ethnicity, we can use insights from these and similar works to appropriately leverage context and semantics to tell more consistent stories in molecular visualization.

Biomedical illustration is a field devoted to illustrating and animating biological and medical topics, often with a human focus. Biomedical illustrators follow perceptual principles in color design of a molecular visualization, but frequently take artistic license regarding the specific colors in a color palette. David Goodsell’s watercolor paintings of molecular machinery are foundational to the practice of illustrating and visualizing molecules, which use color strategically to encode the spatial organization of molecules [3, 22]. However, he notes that the majority of his colors are *“completely arbitrary and are chosen solely for aesthetic appeal* [1].” Biomedical illustrators also frequently employ perceptual color techniques to draw audience attention to the main narrative of the visualization [23]. For example, Jenkinson et al.’s [24] perceptual study on scene complexity versus learning outcomes uses desaturated, low contrast colors for context molecules/scene elements, while applying complementary and highly saturated/bright colors for focus molecules (ligand and protein receptor) in all treatments. Johnson & Hertig suggest the same such approach in their guide to the visual analysis and communication of biomolecular structural data [2]. Wong furthermore notes that small scene objects need increased hue, saturation, and/or brightness to stand out in a visualization [25], and suggests the simple trick of squinting at a visualization to assess for color visibility and evenness. While these approaches are useful for aesthetics and guiding the narrative, none suggest the use of specific colors to semantically highlight particular structural or functional features.

A wealth of publications address the use and efficacy of colormaps, such as the controversial rainbow colormap [26], in visualization. Our concern with color in this work is specific to scientific visualization, and more narrowly to the coloring of molecules and their environments. For an overview of color scales and guidelines for color use in a general visualization context we refer to surveys by Silva et al. [27] and Zhou et al. [28]. Biomedical illustrators and researchers who create molecular visualizations often rely on tools such as ColorBrewer [29], Colourmap Hospital [30], Colorgorical [31], or Adobe Color [32] to determine a color palette for their scene. Many of these tools are designed for chart visualization, as opposed to complex molecular structures in 2D or 3D. Palettes generated from such general–purpose tools can be ineffective or difficult to interpret when applied to molecular visualization. Adobe Color [32] is designed for artists and flexibly allows color selection according to defined color harmony rules, e.g., *complementary* colors. However, this still offers a staggering array of choices and requires a degree of color expertise to use. A similar tool with additional constraints for color selection could be more useful for content authors creating these assets who lack a background in color theory.

Color is used to provide structural cues on a molecular surface. These cues are aided by illumination models, such as Hermosilla et al.’s recent approach [8] that includes realistic diffuse color bleeding over a complete molecular scene. Ambient occlusion and directional lighting are shown by Szafir et al. [9] to help in interpreting molecular surface colors that are in shadow, while stylization can make interpretation more difficult. This study also found luminance-varying ramps to perform better than isoluminant ramps, since shadows reduce the luminance range. This suggests that focus molecules or regions may be better assigned high luminance values to direct attention and improve readability of the molecule’s surface. These works show the importance of selecting appropriate illumination models in a molecular visualization, and should be discussed in future best practices.

Limited works address color application across spatial scales in molecular visualization. Waldin et al. [10] present a technique that employs an systematically adjustable color scheme that mainly relies on hue shift across different levels of magnification, from atomic resolution to a complete virus. Their technique allows the viewer to clearly distinguish between structures of interest at a given level of magnification between, e.g., atoms, domains in a single molecule, or structural compartments of a virus. Colors are generally saturated, and luminance is used as a focus device for particular features, e.g., the quantity of amino acids in a scene, or to show structural details, e.g., secondary structures in a protein domain. This use of luminance to drive a main narrative aligns with conventions in biomedical illustration. However, some instances may require greater contrast than an *analogous* color palette can achieve. Additionally, Waldin et al.’s coloring technique is limited to structure, rather than function. Lastly, the initial molecular color selections made by the user are arbitrary and lack semantic association. Klein et al. [11] apply a similar adaptive multi-scale coloring scheme in the context of microtubule dynamics, where molecules are mainly colored according to structure and have similar arbitrary initial coloring assignments. Developing guidelines from some of the basic rules established in these works, in conjunction with more specific structural or functional coloring rules, could lead to more effective molecular visualizations.

## 3 Color choices in molecular visualization

The colors used to visualize molecules are dependent upon a number of considerations, such as corporate branding, personal taste, and cultural sensitivities. We demonstrate the breadth of color palette choices with a brief sample of color palettes drawn from the Association of Medical Illustrators 2021 Online Salon3
https://meetings.ami.org/2021/onlinesalon/. and from the 2021 VIZBI Poster Gallery4
https://vizbi.org/Posters/. in the Proteins category.

### 3.1 Color considerations

Numerous factors influence the choice of a molecular color palette. Pharmaceutical marketing and research are major drivers for the production of molecular visualizations. Marketing videos for a new drug are contracted to specialty biomedical illustration studios every year. In many of these instances, the brief requests color palettes to follow the branding of the drug or the parent pharmaceutical company. In other instances the client may focus less on brand colors but instead request a palette reflective of a particular mood, e.g., comforting or dramatic. Such client–specific color requests can be helpful in constraining a design space that is at times overwhelming, but are partially responsible for the broad range of palettes and the lack of semantic consistency in the coloring of particular molecules. Beyond the pharmaceutical sector, different target areas often play a role in color palette choices. A molecular visualization aimed at academic/educational use often drives the author to make different color choices than a visualization for pharmaceutical use, as the main goal for this sector is often to engage and teach a broad, diverse audience. For example, Drew Berry’s work mainly targets the educational and research sector, with visualizations for colorblind-friendliness and frequent use of yellows, blues, and purples applied to lambert shaders with ambient occlusion [33]. In contrast, XVIVO Scientific Animation studio [34] tends towards more high-end, flashier lighting, shading, and rendering techniques, which contribute to color palettes with greater drama and contrast for clients in the biotech and pharmaceutical sectors.

Culture is also a major driver of color selection, given its affective role in visualization. Many molecular visualizations incorporate blue, or a close *analogous* color, into their palette for the comforting, pleasant emotions that often associate with this color [12, 35]. Various works have shown that cool colors, e.g., blue, green, are more passive than warm colors, e.g., red, yellow, and orange [6, 12, 16, 36], and thus can be good context color choices. While the color red is an active, i.e., highly visually salient color [12, 36], depending on the culture it can indicate danger or, conversely, luck or happiness according to Chinese culture.5
https://informationisbeautiful.net/visualizations/colours-in-cultures/. Biomedical illustrators based in North America commonly use red to indicate aberrant molecular activity, e.g., constitutive activation, where a molecule is always turned “on.” For East Asian cultures this is not semantically intuitive, and may be taken to mean the opposite for audiences that do not have this exposure to North American conventions.

Likely the most significant element of color choice for molecular visualizations comes down to the author’s tastes and aesthetic preferences. Furthermore, biomedical illustrators and studios often wish to develop a house style that sets them apart from other studios. The decision process for color selection may often be guided by basic perceptual principles, e.g., saturated focus and desaturated context, and color harmony rules. However, the ultimate decision to color a ligand purple, orange, or another color is the author’s decision with little to no semantics attached. Exceptions include the case mentioned previously in a North American context, where red is often used to indicate aberrant activity of a molecule [37], and the use of red to color hemoglobin, which is the oxygen-bearing protein in red blood cells [22]. However, the majority of molecular visualization color palettes are guided by the author’s aesthetic sensibilities and their storytelling goals. The visualizations produced by Drew Berry, such as his set of DNA animations [38], are one example of this, where color application is highly aesthetic and tells a clear story, but the coloring of the individual molecules does not necessarily tie to their respective structural or functional properties.

### 3.2 Color strategies: a contemporary sample

To illustrate the broad use of color in practice for molecular visualizations today, we conducted an informal study where we extracted the color palettes from 20 molecular visualizations that were produced in the last year. This ensures that our sampling captures recent trends in color design. Since such works may be created by biomedical illustrators, bioinformaticians, structural biologists, and visualization researchers [37], we sampled from two venues that attract these professions, and sampled ten palettes from each. The Association of Medical Illustrators (AMI) is a global, although primarily oriented to North America, society of biomedical illustrators. Every year the association hosts a juried salon where student and professional work can be submitted, and which is subsequently posted online. The ten palettes we sampled from the salon featured works where molecules were the main story element, and came from either static images or stills from a larger animation. Visualizing Biological Data (VIZBI) is an annual meeting that brings together diverse professions to discuss advances in research in biological data, including molecular data. Each year includes a large poster session, which is divided into topical categories. The ten palettes we sampled came from the 2021 Proteins poster category. While VIZBI is advertized as an international venue, we acknowledge a North American bias in our sampling of biomedical illustration work from the AMI, since this is a primarily North American-oriented organization.

We generated color palettes from each molecular visualization using the Adobe Color tool [32], which has a feature to extract a color palette from an image. Palette extraction can be done manually by user selection of pixels of interest in the image, or semi-automatically according to *color mood*, e.g., *colorful* or *muted*. We used this semi-automatic feature as a way to minimize subjectivity bias for this phase. Experimentation with each of the five color mood options found that *colorful* consistently yielded palettes that captured the broadest color range in the image and most often included colors of molecules that may have proportionally occupied fewer pixels but were important to include in terms of the story. Palettes from this mode were the closest to what we, as experienced authors of molecular visualizations, would have generated by eye. However, in some instances the system did not pick up an important story feature. In these instances we manually adjusted the palette, replacing a redundant color, e.g., if the generated palette included two shades of a color, to include this color instead. This manual adjustment to the palette introduces a possible subjective bias in the study design.

After a palette is generated from an image, Adobe Color stores the resulting color palette as only a “custom” color harmony rule. Our intervention was required to match this custom palette to the closest-matching harmony rule, which we did by attempting to duplicate the palette by eye for each rule setting. This involved at times changing what the system had identified as the central color, or by making slight adjustments to a palette color to identify the harmony rule that was the closest, if not exact, match for the generated palette. This human subjectivity is another possible source of bias, although we did our best to limit this through consistency of choices and monitor use. We further discuss this possible bias in Section 5.

**Figure 5:**
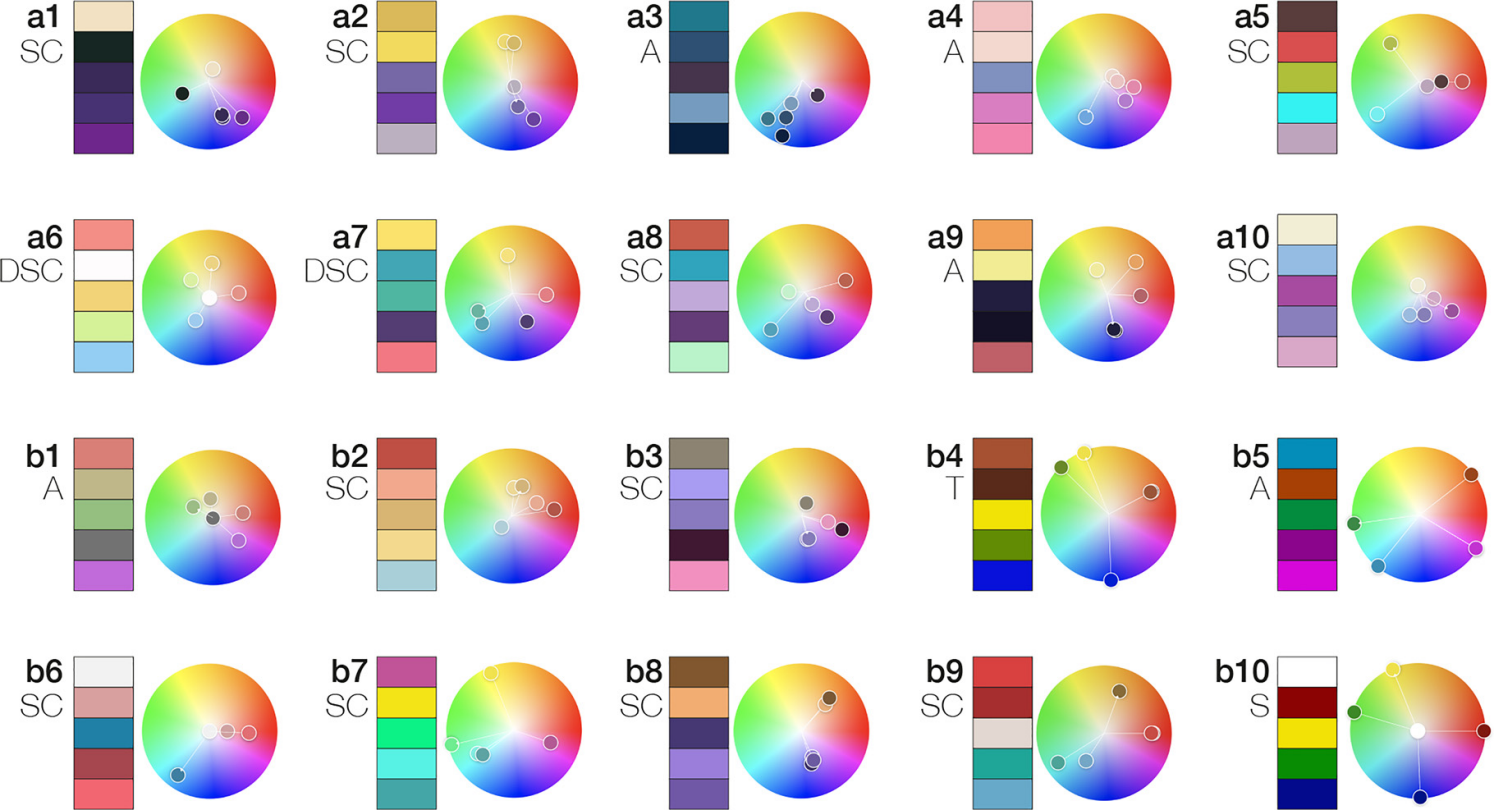
20 sampled molecular visualization color palettes. (a) 2021 AMI Online Salon color palettes. (b) 2021 VIZBI Protein poster color palettes. *Rule abbr: SC, split complementary; DSC, double split complementary; A, analogous; T, triad; S, square.*

Figure 5 shows the set of 20 palettes that we generated from this process. Palettes a1–a10 are sourced from the AMI Salon, while b1–b10 are sourced from VIZBI Protein posters. For further details on the content authors, title and link to their original work, color harmony rules, and resulting palettes we refer to Supplementary Material. Although palettes are not always a precise match to a given harmony rule, we were able to match all 20 to a closest harmony rule. *Split complementary* is the most common rule, employed in 11 palettes in both groups, with two additional *double split complementary* palettes used in the AMI group. Palettes with an *analogous* rule are the second most common (five), and occur more frequently in the AMI group. The VIZBI group exhibits harmony rules that are closest to a *triad* (b4) and a *square* (b10) rule.

Generally, the palettes generated from the AMI group tend to be less saturated, show a greater contrast range, and favor *split/double split complementary* rules in their palettes. VIZBI group color rules for palettes are more variable, although *split complementary* is the dominant rule for this group as well. This latter point could reflect that some of the posters submitted were created in part by biomedical illustrators, e.g., b1 and b2. Cool base colors (purple, green, or blue) are more common (11) relative to warm colors, but this is fairly evenly–balanced between both groups.

Although we did not observe consistent color semantics across all 20 visualizations, some patterns emerge. We discuss these in the following, but note the need for a larger, international study before using such findings as the basis for formal and actionable guidelines. VIZBI posters that visualize COVID-19 more consistently use red for the spike proteins (b2, b6, b9), although this is not true for all (b4). The AMI group visualization of COVID-19 (a4), while using a warm color, uses pink instead. Purple is a popular color for receptor proteins for the AMI group (a2, a3, a5, a8), although also used for membrane molecules (a7, a8, a10), ligands (a4), or other elements (a9). VIZBI posters use purple less frequently, and more often for a ligand (b1, b5, b7) rather than for a receptor (b3) or other structural element (b8). In comparison, AMI works favor yellow/orange for a ligand (a1, a2, a3, a8, a10) or other focus molecule (a6, a7, a9). Yellow/orange is used more broadly, and less frequently, in the VIZBI group. These findings show that, while color selection on the whole is largely arbitrary, there is a clear aspect of peer influence in color selection, particularly in the AMI group. Best practices could be formalized from this, particularly if a larger survey with international groups and cultures uncovers a similar and stronger pattern of color application. In both groups on a per-visualization basis, individual molecular coloring is consistent for structural elements, e.g., molecules comprising a cell membrane are all colored the same or *analogously*. However, the semantics behind the color choices are unclear, except in the case of the COVID-19 spike protein’s red coloring. Emphasizing a visual hierarchy or to focus attention on the main narrative appears to be the primary factor in color selection, with only sporadic consistency in coloring particular types of molecules, e.g., ligands.

## 4 Considerations for molecular coloring best practices

Molecular visualization remains a young and rapidly growing field. Allowing room for creativity is important for biomedical illustrators, structural biologists, and experts from related fields to innovate on the ways that we visualize molecules. However, framing coloring approaches within a set of best practices can provide a common ground that aids in the **aesthetics**, **interpretability**, and ultimate **effectiveness** of a molecular visualization while also simplifying the design process for content authors with limited training in color theory.

**Figure 6:**
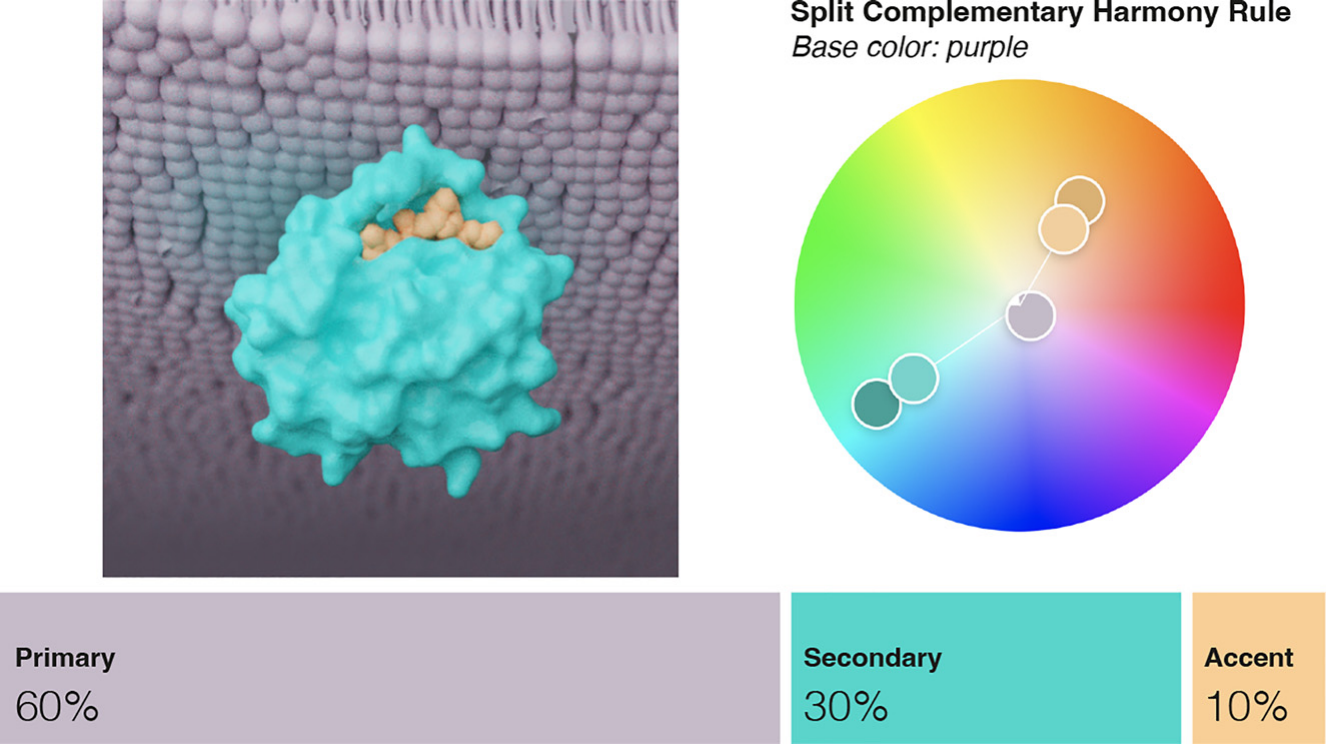
Example of 60-30-10 rule used in a biomedical illustration. Explorable color palette at https://color.adobe.com/color-name_LG-color-theme-19646985/.

**Aesthetics.** Aesthetics are integral to drawing and guiding attention in a molecular visualization. Best practices can help content authors who lack formal training or intuition in color theory to easily craft more aesthetically–pleasing visualizations. The *60-30-10* rule from interior design is a useful rule of thumb to guide the composition and harmony of a molecular visualization. In this rule, 60% of the scene should be a dominant color, 30% a secondary color, and the last 10% an accent color, as demonstrated in Figure 6. The *split complementary* harmony rule that is popular with biomedical illustrators aligns with this practice. Molecular visualizations that follow established color harmony rules and basic perceptual principles can be more aesthetically–pleasing, and furthermore may be easier to interpret in their ability to guide user attention to salient story points [2, 23, 25].

**Interpretability.** Color is an important driving force in the interpretability, or readability, of a molecular visualization. It can help the audience focus on the intended parts of the story, which ultimately leads to a more effective visualization. For instance, readability guidelines for text-based presentations recommend roughly 80% contrast between focus and context elements [39]. Additionally, employing *active* versus *passive* color ranges can clearly define focus + context elements and establish a scene hierarchy. Although different cultures or other contexts may assign different semantic meanings, warm, saturated, or light value colors perceptually tend to draw attention, while cool, desaturated, dark value colors often recede visually when positioned with active colors [6]. This perceptual feature is already in broad use in biomedical illustration, although, as we observed in our small study, the specific colors themselves are inconsistent. Leveraging this natural perceptive feature can aid interpretability of a molecular visualization by using color salience to draw attention to the most important, i.e., focus elements, of a visualization. For example, ligands and their receptors can be assigned high contrast colors relative to context molecules. *Complementary* colors can then be employed to differentiate the ligand from its receptor. A *split complementary* palette of yellow (ligand) and purple (receptor), such as in Figure 5a2, is a colorblind–friendly choice for clear differentiation of ligand and receptor. Molecules that comprise a pathway can similarly be colored for high contrast against context elements, and these pathway molecules could be *analogously*-colored to indicate functional relatedness, as in Figure 2. Importance functions could be useful for rule-based methods to aid in generating a molecular visualization and assigning appropriate hue, saturation, and lightness values to assets.

**Effectiveness.** Lastly, an effective molecular visualization is read *correctly* by the intended audience. For example, in a given visualization, can the audience correctly identify a ligand? Coloring best practices can help to create a semantic layer of communication that provides an intuition to a broad audience of certain structural or functional properties of a molecule. This is particularly valuable since molecules are themselves rather abstract, often looking like “partially-melted gummy bears” that are difficult to relate to macroscale-world structures. Just as how a lay audience may not necessarily know exactly what a red blood cell is or what it does at a technical level, through shape and color cues they can recognize its basic properties and relate it to blood on a larger scale. This strategy can be extended to the molecular level by coloring hemoglobin red. Consistent coloring across multiscale may facilitate understanding of properties of other molecules as well.

Coloring molecules according to the type of pathway that they are involved in is another consideration. Certain color families and harmony rules could be applied to, e.g., signal transduction pathways, while metabolic or gene expression pathways are assigned a different color family. Further color treatment in the form of different applications of color fresnel or glow effects could then be used if the pathway is showing aberrant activity, as is frequent in cancer. This guideline remains general enough to allow for creative license and follows principles for aesthetic and interpretable visualizations, while adding additional semantics for greater understanding.

An alternative consideration could be assigning color families and harmony rules according to structure. This could be related to the structure of a single molecule, e.g., the domains of a protein could be assigned particular shades or tints. This guideline may also be applied to the structure of entire molecular scenes, e.g., membrane structures that are comprised of thousands of phospholipid molecules is assigned to a particular color family, while native internal molecular structures are allocated to a different portion of the color space. Currently, coloring by structure is mostly consistent within a given visualization, but this is not the case when comparing between the majority of visualizations. Coloring according to structure has some natural overlap with coloring according to function. Guidelines for both perspectives could be applied together to give color greater meaning in a molecular visualization.

Lastly, cultural associations are important considerations in developing guidelines to ensure the semantics come across as intended. In our globalized world, adoption of best practices that generalize across cultures is preferable to, e.g., palettes biased towards Western sensibilities. Works such as Adams et al.’s [12] cross-cultural study of the affective meanings of color are a useful starting point. The development of guidelines for coloring of molecular visualization that help achieve these three aims may also draw inspiration from works on color specification models for scientific data, such as by Nardini et al. [40].

## 5 Limitations

This work presents a discussion and limited study of the space of color choices used in industry and research, and additionally draws on the authors’ own experience in crafting molecular visualizations. While our aim is to summarize and introduce considerations for developing guidelines and best practices for semantically meaningful molecular color palettes, we note some limitations in this paper.

The color palettes selected in our study and the rationales we discuss for authors’ color palette selection draw primarily from North American or European venues and motivations. This bias is important to note, and acts as further motivation to conduct larger, international studies and focus groups for the formal development of best practices. We do not comprehensively summarize the various rationales for color palette selection, and rather discuss a subset of the most common rationales. Similarly, we do not aim to comprehensively summarize the space of color usage in molecular visualization. This is an interesting direction and may be useful in the establishment of robust guidelines, but is beyond the scope of this work. Although limited, our considerations are meant to provide a starting point for establishing and discussing the need for best practices in coloring molecular visualizations.

A second source of possible subjectivity and bias is the generation of color palettes from images that we sampled in our study. Although Adobe Color is a high-quality tool popular in both research and industry for color palette creation, use of another tool may lead to variations in the color palettes that we generated. Our decision to use a particular color mood and subsequent minor adjustments we made to determine color palettes and their closest harmony rules were based on the subjective opinion of the researcher generating the color palette. Additionally, our criteria for adjusting automatically-generated palettes to include a color important to the story is another source of subjectivity in palette generation. This could have had an effect on the resulting harmony rule. Bias could have been mitigated in these instances by having another researcher generate palettes and compare the results for consistency. However, we did not incorporate this into our pipeline, as this is a preliminary study to explore color use and to motivate future work in this space.

Despite the fact that we frame our discussion of color in this paper around the HSL color space, this may not be the optimal color space. Although spatially uniform and therefore easier to describe, it is not perceptually uniform. For the latter, a color space such as HCL (hue, *chroma*, and lightness) [41, 42] may be a more suitable alternative.

The spatial range that molecular visualization covers is large, spanning from tiny individual molecules up to large scenes of molecules that form even larger structures. An extensive discussion of the multiscale nature of the molecular world falls beyond the scope of this work, but this is a major challenge that best practices for coloring molecular visualizations must account for. Audience tasks may also be different at each scale. For example, CPK coloring rules are useful to identify residues for binding sites, but a different rule is necessary when exploring protein secondary domains, or an entire cell at molecular resolution. Works such as Waldin et al. [10] provide a solid foundation for best practices in coloring across spatial scales.

## 6 Challenges & outlook

Establishing a robust set of guidelines that retain the flexibility for creative expression and innovation is challenging and requires further research in the form of perceptual user studies. For example, Jenkinson et al. [24] present a perceptual study that assesses the effect of molecular scene complexity on learning outcomes for undergraduate biology students. Presenting four scenes of increasing complexity that depict protein-ligand binding conformational changes in 3D, the authors found that the most complex scene was the most effective in achieving the intended learning outcomes. A similar study design could be adopted to assess the effects of different color palettes on learning outcomes.

Focus groups on color use with experts in biomedical illustration, visualization, structural biology, and bioinformatics are also necessary when developing guidelines to ensure that all stakeholders have a voice in the process. Our prior work has explored stakeholder perspectives from biomedical illustration and animation studios and visualization researchers [37]. This was a limited study that centered on North American and European molecular visualization conventions, and obtained these stakeholder perspectives through 1:1 interviews and in small focus groups (3–4 people). Such qualitative research methods should be used for the development of more formal guidelines, which we envision could take place within a workshop format that invites stakeholders from all relevant disciplines to achieve adequate representation. The Creative Visualization-Opportunities (CVO) Workshop [43] is a promising model to follow to ensure diverse stakeholder participation and engagement.

Colors are not interpreted in the same way in all cultures. Guideline development requires numerous, inclusive discussions and perceptual studies with people from different backgrounds. Further, international studies into the use of color and its meaning beyond Western aesthetics are necessary. As previously discussed, CVO workshops [43] that recruit stakeholders not only from a diversity of professions but also a diversity of cultures, genders, and ethnicities are imperative to developing guidelines that are globally relevant. Avoiding pitfalls related to color blindness is another challenge. For example, red-green color blindness is well-known and accounted for in standard design practice. Deciding to color hemoglobin red to enable its semantic mapping to red blood cells and blood can be problematic if green is also a part of the visualization’s palette. Further nuanced discussion of affective and perceptual conflicts is necessary.

The use of color in the context of glows, as demonstrated in Figure 1b, also requires discussion. Glows are notoriously ambiguous in their semantics: they can indicate generic activity, aberrant activity, metabolism, growth, pain, among others. Conducting studies exploring the use and meaning of glows in the context of molecular events is likely the best way forward to determine best practices for their use (or disuse).

## 7 Conclusions

Current color palettes for molecular visualizations are largely arbitrary and determined by a wide range of factors. Implementing coloring best practices can lead to more interpretable and effective molecular visualizations that are stylistically and semantically consistent, without overly compromising on creative freedom or aesthetics. Such guidelines can also simplify the color design process. These guidelines could be applicable not only for communication-oriented tasks, but for exploratory and analytically-oriented visualizations as well. Color can be a means to elevate scientific and health literacy at individual and population levels. With molecular visualization becoming more prevalent in mainstream culture, we feel that color best practices can, and should, be implemented to improve public understanding of molecules.

## Supplementary Material

Supplementary Material Details
