# Supplementary material for: Considering best practices in color palettes for molecular visualizations
Source: J Integr Bioinform. 2022 Jun 22;19(2):20220016. doi: 10.1515/jib-2022-0016 (PMC9377702; doi:10.1515/jib-2022-0016)
Supplement: Supplementary file 1 — Supplementary Material Details [file j_jib-2022-0016_suppl.zip › supp_material/index.html]

Molecular Color Palettes Database  

# Molecular Color Palettes Database

| ID | Visualization Title | Author | Harmony Rule (Nearest) | Base Color | Color Theme URL | Image | Venue | Visualization Source URL | Additional Notes |
| --- | --- | --- | --- | --- | --- | --- | --- | --- | --- |
| a1 | **To Kill Cancer, Turn Off the Power** | Katie Harvey | Split complementary | Purple | https://color.adobe.com/color-theme\_a1-color-theme-19638529/ |  | AMI 2021 Online Salon | https://meetings.ami.org/2021/project/to-kill-cancer-turn-off-the-power/ | split complementary color scheme, base color purple |
| a2 | ****Window Chamber Method: A Novel Approach of Cellular Imaging**** | Su Min Suh | Split complementary | Yellow | https://color.adobe.com/color-theme\_a2-color-theme-19638526/ |  | AMI 2021 Online Salon | https://meetings.ami.org/2021/project/window-chamber-method-a-novel-approach-of-cellular-imaging/ | split complementary, base color yellow |
| a3 | ****The Science of Neural Communication**** | Katherine Knack, Michael Astrachan(XVIVO) | Analogous | Blue | https://color.adobe.com/color-theme\_a3-color-theme-19638523/ |  | AMI 2021 Online Salon | https://meetings.ami.org/2021/project/the-science-of-neural-communication/ | analogous, base color blue |
| a4 | ****SARS-CoV-2 G614 Mutant Increases ACE2 Receptor Binding**** | Nicole Shepherd | Analogous | Purple | https://color.adobe.com/color-theme\_a4-color-theme-19646865 |  | AMI 2021 Online Salon | https://meetings.ami.org/2021/project/sars-cov-2-g614-mutant-increases-ace2-receptor-binding/ | analogous, base color fuschia |
| a5 | ****Omega Therapeutics Epigenomic Programming MOA**** | David Ehlert, Inessa Stanishevskaya, Brandon Keehner | Split complementary | Purple | https://color.adobe.com/color-theme\_a5-color-theme-19646864 |  | AMI 2021 Online Salon | https://meetings.ami.org/2021/project/omega-therapeutics-epigenomic-programming-moa/ | split complementary, dusty red/purple base color |
| a6 | ****BiTE® Immuno-oncology platform**** | Jason Sharpe, Eddy Xuan, Lima Colati (AMGEN) | Double split complementary | Red tintWhite | https://color.adobe.com/color-theme\_a6-color-theme-19646862 |  | AMI 2021 Online Salon | https://meetings.ami.org/2021/project/bitea-immuno-oncology-platform/ | double split complementary (closest), base color dusty white with red tint |
| a7 | ****C\_TheScience**** | Jason Sharpe, Eddy Xuan, Lima Colati (AMGEN) | Double split complementary | Green | https://color.adobe.com/color-theme\_a7-color-theme-19646860 |  | AMI 2021 Online Salon | https://meetings.ami.org/2021/project/c\_thescience/ | double split complementary, base green |
| a8 | ****View into a Synapse to Feature the Release of GABA, and its Binding to the Pentameric GABA Receptors**** | Audra Geras | Split complementary | Purple | https://color.adobe.com/color-theme\_a8-color-theme-19646859 |  | AMI 2021 Online Salon | https://meetings.ami.org/2021/project/view-into-a-synapse-to-feature-the-release-of-gaba-and-its-binding-to-the-pentameric-gaba-receptors/ | split complementary, base purple |
| a9 | ****NLRP3 Inflammasome Assembly**** | Veronica Falconieri Hays | Analogous | Pink | https://color.adobe.com/color-theme\_a9-color-theme-19646853 |  | AMI 2021 Online Salon | https://meetings.ami.org/2021/project/nlrp3-inflammasome-assembly/ | analogous, base color salmon pink |
| a10 | ****A Binding Proposal: How is NOD’s proposed bacterial glycan-binding site related to Crohn’s Disease**** | Martin Shook | Split complementary | Yellow | https://color.adobe.com/color-theme\_a10-color-theme-19646832 |  | AMI 2021 Online Salon | https://meetings.ami.org/2021/project/a-binding-proposal-how-is-nods-proposed-bacterial-glycan-binding-site-related-to-crohns-disease/ | split complementary (closer to this than analogous), base color yellow to achieve harmony rule |
| b1 | ****Visualization of G Protein-Coupled Receptor Cis-Membrane Diffusion**** | J. Hornby, B. van Rossum, S. Thapa, R. Metzler, & G. McGill | Analogous | Green | https://color.adobe.com/color-theme\_b1-color-theme-19646828 |  | VIZBI 2021 Protein Poster | https://vizbi.org/Posters/2021/vD14 | analogous scheme (closest), base color green |
| b2 | ****Capturing the SARS Spike in motion – visualizing a molecular contortionist**** | Gael McGill | Split complementary | Blue | https://color.adobe.com/color-theme\_b2-color-theme-19646826 |  | VIZBI 2021 Protein Poster | https://vizbi.org/Posters/2021/vD25 | split complementary, base color blue |
| b3 | ****Blood-Brain Barrier**** | Martina Fröschl | Split complementary | Pink | https://color.adobe.com/color-theme\_b3-color-theme-19646825 |  | VIZBI 2021 Protein Poster | https://vizbi.org/Posters/2021/vC06 | split complimentary, base color pink |
| b4 | ****Macromolecular Complexes Visualizations at Atomic Resolution**** | Victor Padilla-Sanchez | Triad | Brown | https://color.adobe.com/color-theme\_b4-color-theme-19646823 |  | VIZBI 2021 Protein Poster | https://vizbi.org/Posters/2021/vC02 | triad (closest), base color brown |
| b5 | **PDB Component Library** | PDB Component Library Collaboration, Presented by Mandar Deshpande (EMBL-EBI) | Analogous | Purple | https://color.adobe.com/color-theme\_b5-color-theme-19646819 |  | VIZBI 2021 Protein Poster | https://vizbi.org/Posters/2021/vD13 | analogous scheme (broad range), base color is purple |
| b6 | ****PathogenAR**** | Kristen Browne | Split complementary | Red tintWhite | https://color.adobe.com/color-theme\_b6-color-theme-19646817 |  | VIZBI 2021 Protein Poster | https://vizbi.org/Posters/2021/vC19 | split complementary (close), base color dusty white with red tint |
| b7 | ****Gold Nanoparticle-based Artificial Nucleases on the Move**** | Adam Pecina, Laura Riccardi, Paolo Scrimin, Fabrizio Mancin and Marco De Vivo | Split complementary | PinkPurple | https://color.adobe.com/color-theme\_b7-color-theme-19646807 |  | VIZBI 2021 Protein Poster | https://vizbi.org/Posters/2021/vB24 | split complementary (close), base color purple to achieve the harmony rule |
| b8 | ****Learning from Static and Dynamic Animations in Undergraduate Molecular Biology Education**** | Aygen Ergen, Salih Ofluoglu | Split complementary | White | https://color.adobe.com/color-theme\_b8-color-theme-19646803 |  | VIZBI 2021 Protein Poster | https://vizbi.org/Posters/2021/vA22 | split complementary, base color white |
| b9 | ****Fantastic Voyage in VRCell World**** | Daisuke Inoue | Split complementary | BlueGreen | https://color.adobe.com/color-theme\_b9-color-theme-19646799 |  | VIZBI 2021 Protein Poster | https://vizbi.org/Posters/2021/vB03 | split complementary (close), base color blue/green to achieve the harmony rule |
| b10 | ****NAChRDB: Interactive annotations to untangle allostery of nicotinic acetylcholine receptor**** | Aliaksei Chareshneu, Purbaj Pant, Ravi José Tristão Ramos, David Sehnal, Tuğrul Gökbel, Crina-Maria Ionescu, & Jaroslav Koča | Square | White | https://color.adobe.com/color-theme\_b10-color-theme-19646783 |  | VIZBI 2021 Protein Poster | https://vizbi.org/Posters/2021/vA01 | square (closest), angles not exactly 180 but general idea of green/red and blue/yellow is there |
