# Supplementary figures and images for: Considering best practices in color palettes for molecular visualizations
Source: J Integr Bioinform. 2022 Jun 22;19(2):20220016. doi: 10.1515/jib-2022-0016 (PMC9377702; doi:10.1515/jib-2022-0016)

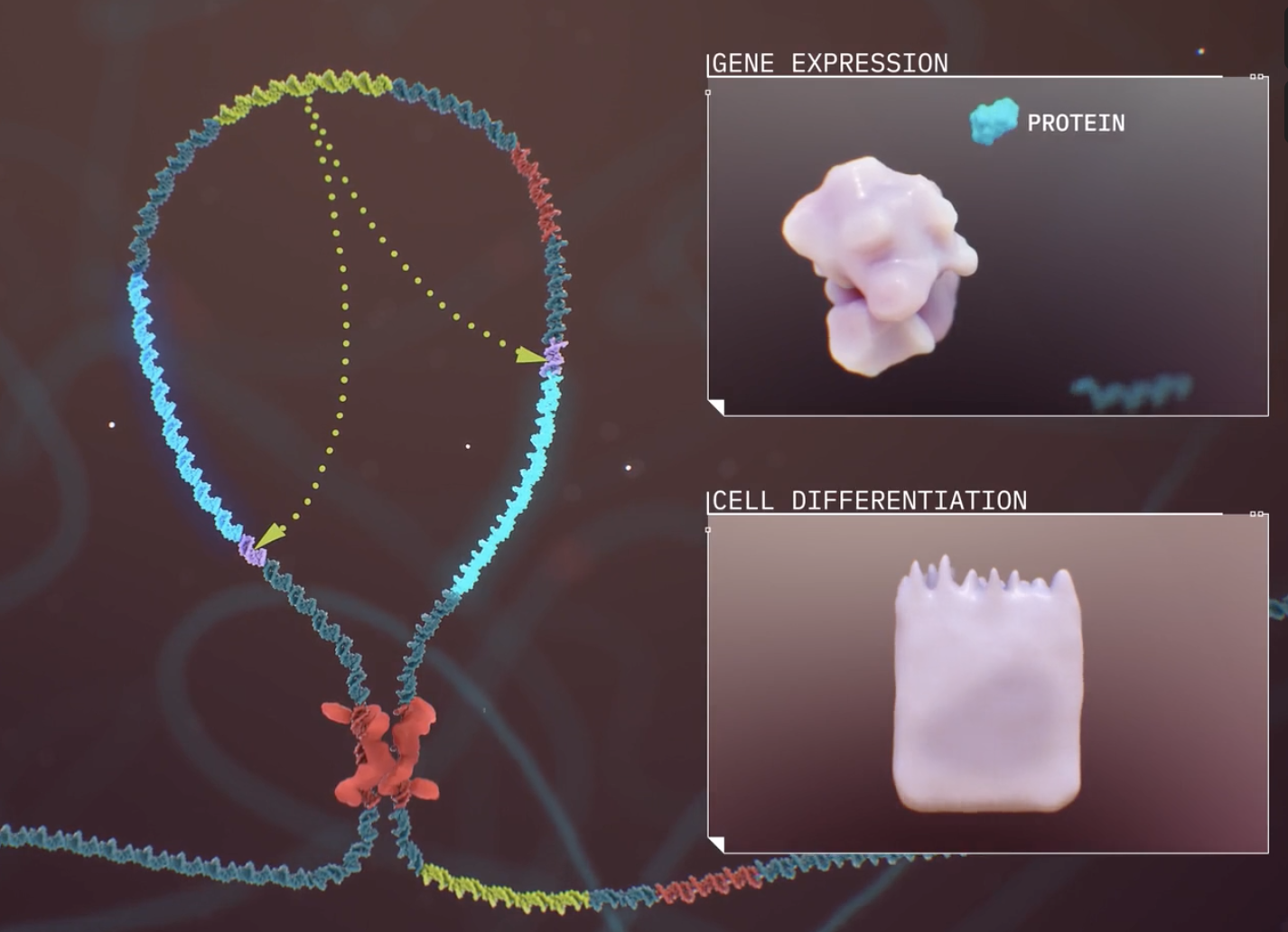

Supplement: Supplementary file 1 — Supplementary Material Details [file j_jib-2022-0016_suppl.zip › supp_material/img_src/David Ehle f99d4/Screenshot_2022-03-10_at_08.53.27.png]

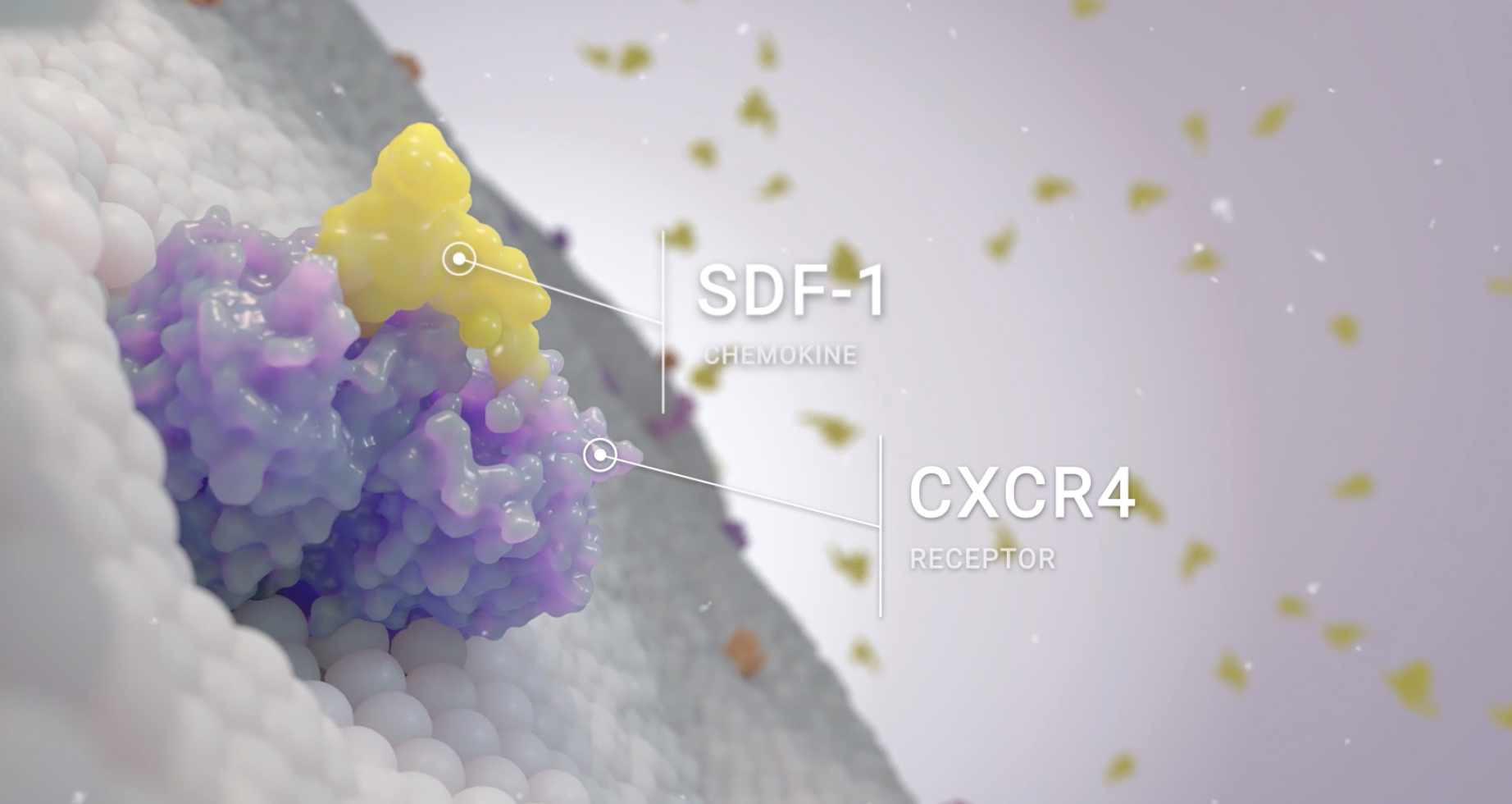

Supplement: Supplementary file 1 — Supplementary Material Details [file j_jib-2022-0016_suppl.zip › supp_material/img_src/Su Min Suh 27208/Screenshot_2022-02-28_at_09.53.07.png]

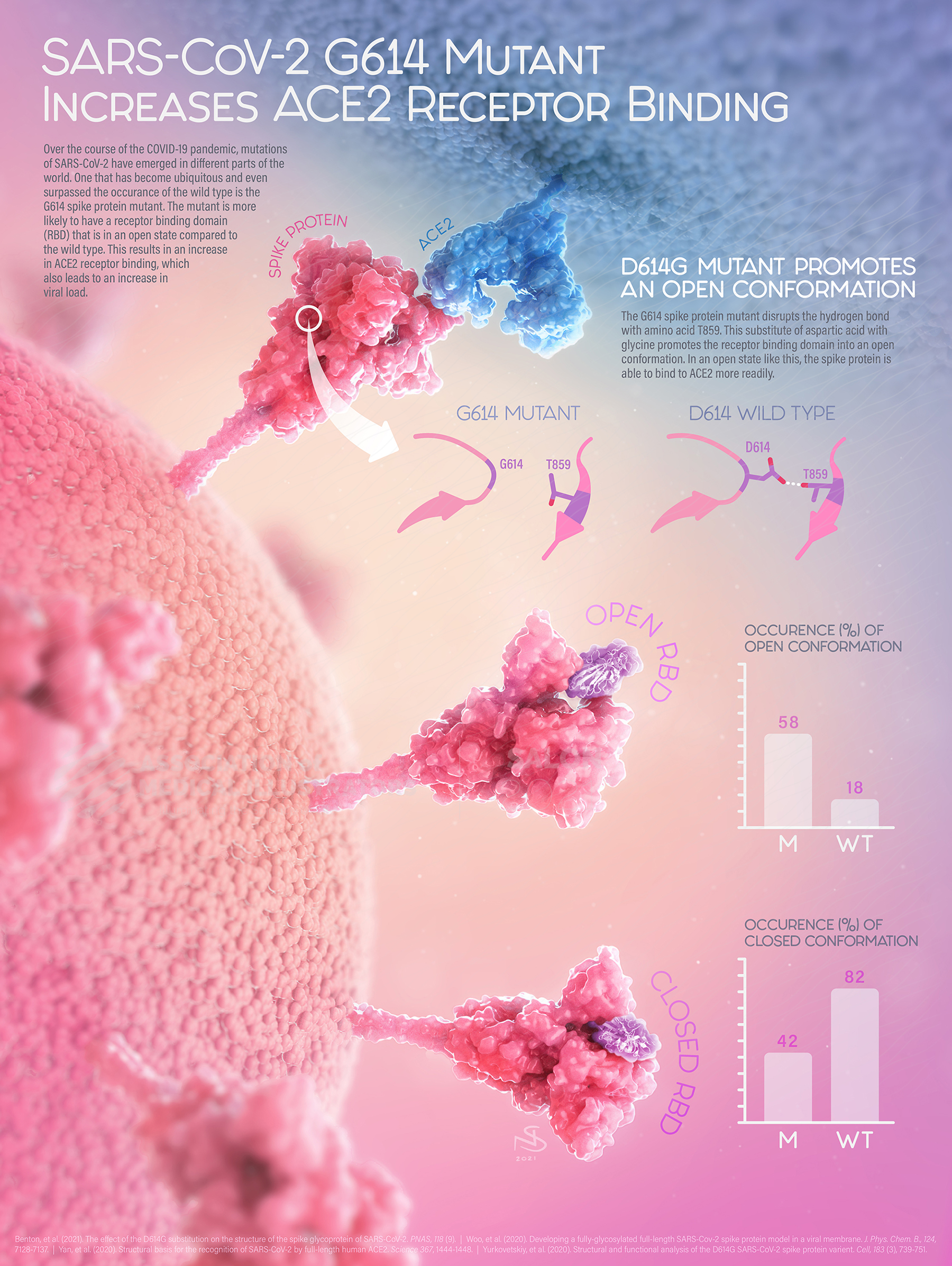

Supplement: Supplementary file 1 — Supplementary Material Details [file j_jib-2022-0016_suppl.zip › supp_material/img_src/Nicole She e173f/Nicole-Shepherd-I3_SARSCov2G6.jpeg]

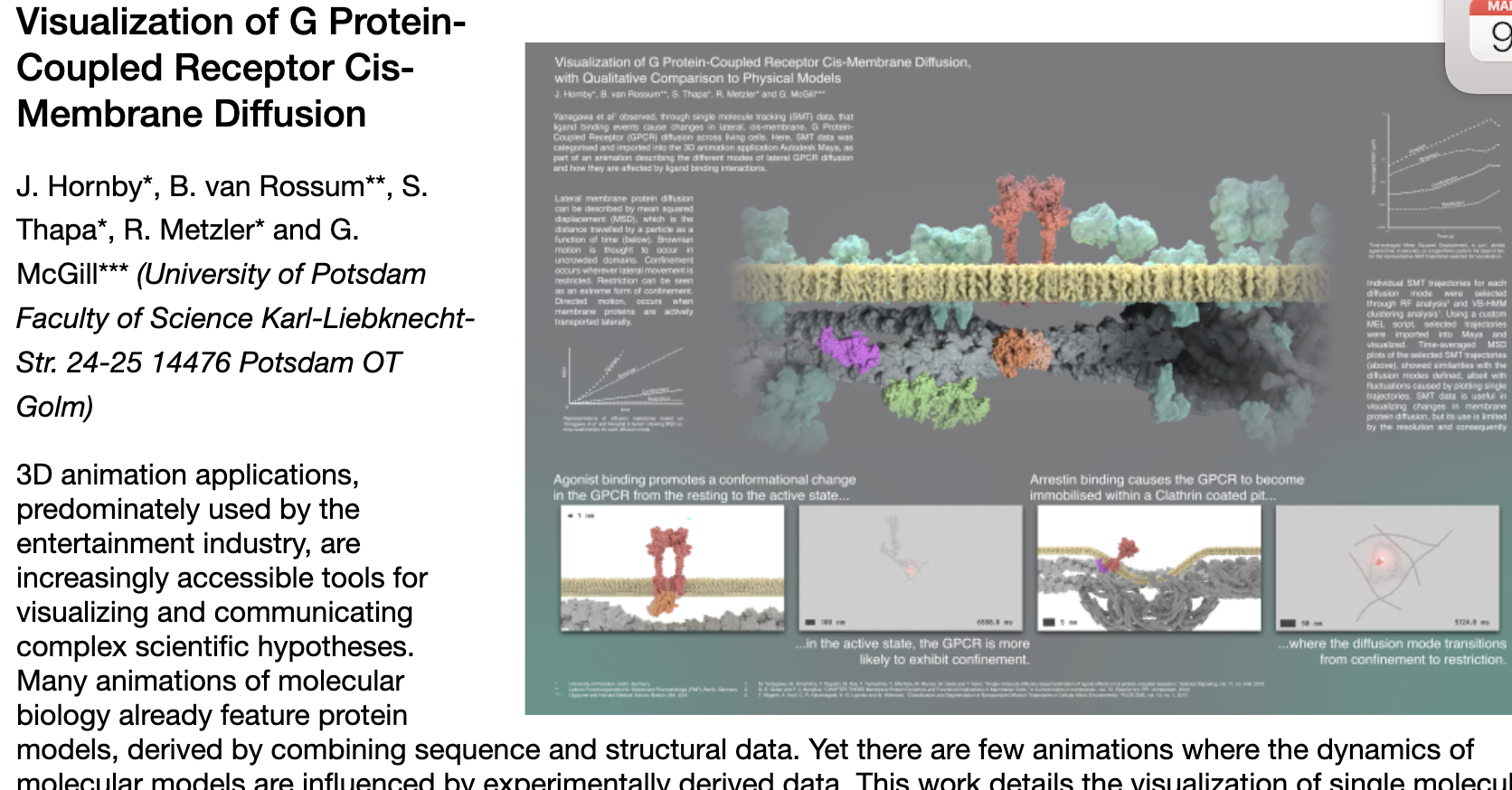

Supplement: Supplementary file 1 — Supplementary Material Details [file j_jib-2022-0016_suppl.zip › supp_material/img_src/J Hornby, 0b0eb/b1.png]

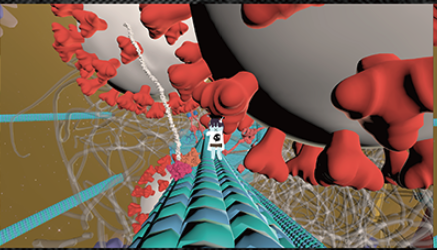

Supplement: Supplementary file 1 — Supplementary Material Details [file j_jib-2022-0016_suppl.zip › supp_material/img_src/Daisuke In ea272/b9.png]

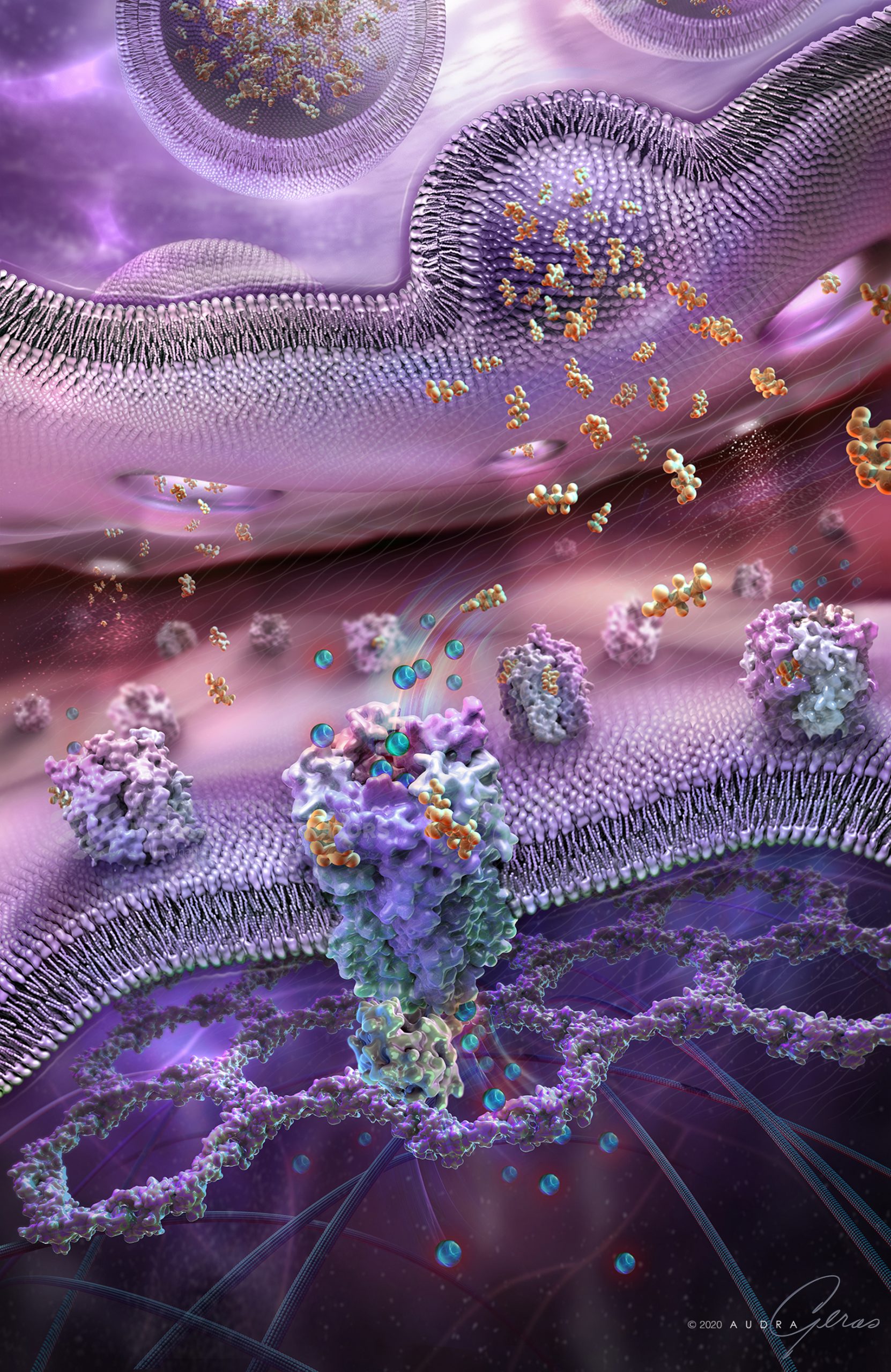

Supplement: Supplementary file 1 — Supplementary Material Details [file j_jib-2022-0016_suppl.zip › supp_material/img_src/Audra Gera e4d9d/a8.jpeg]

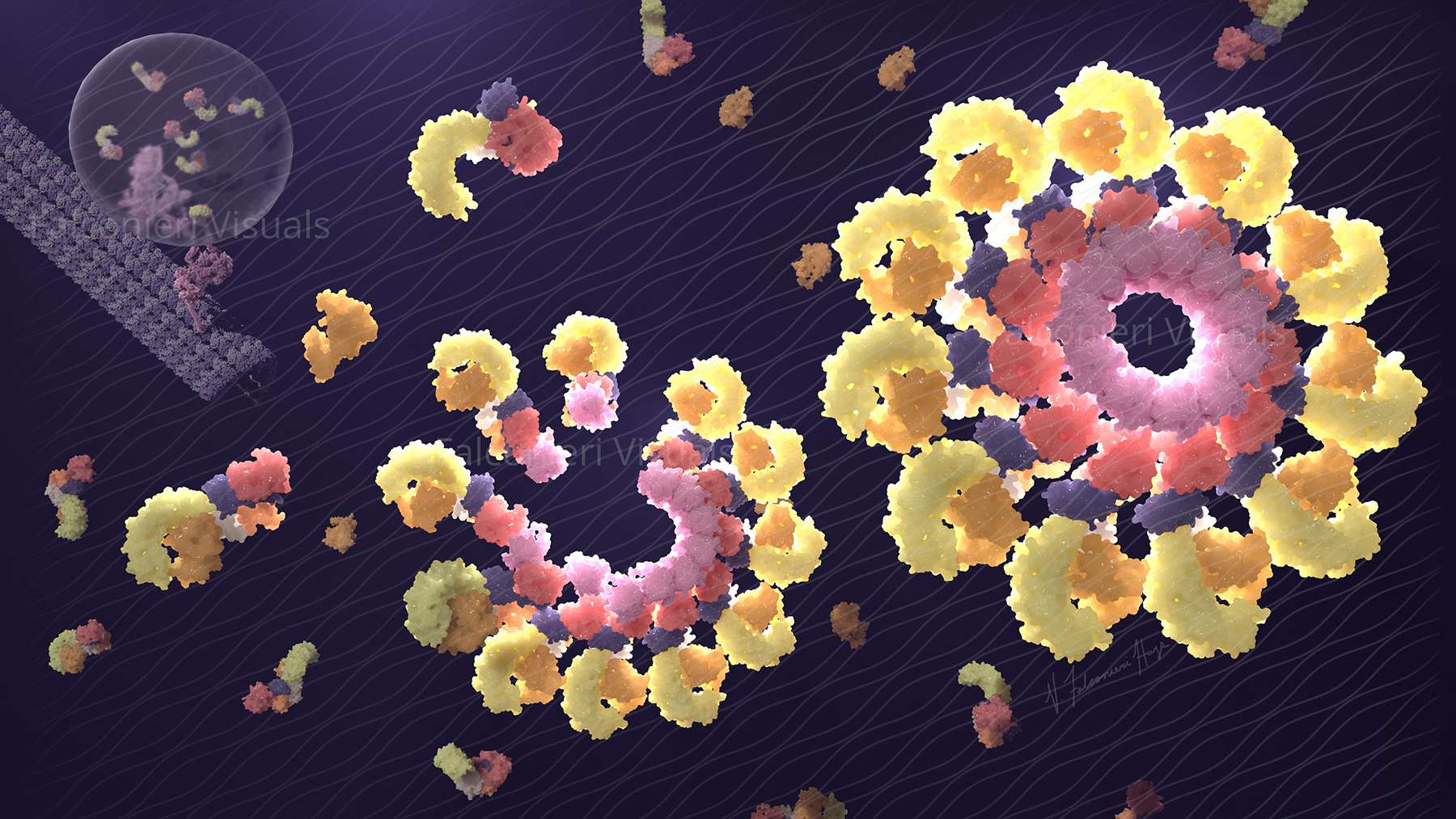

Supplement: Supplementary file 1 — Supplementary Material Details [file j_jib-2022-0016_suppl.zip › supp_material/img_src/Veronica F b0467/a9.jpeg]

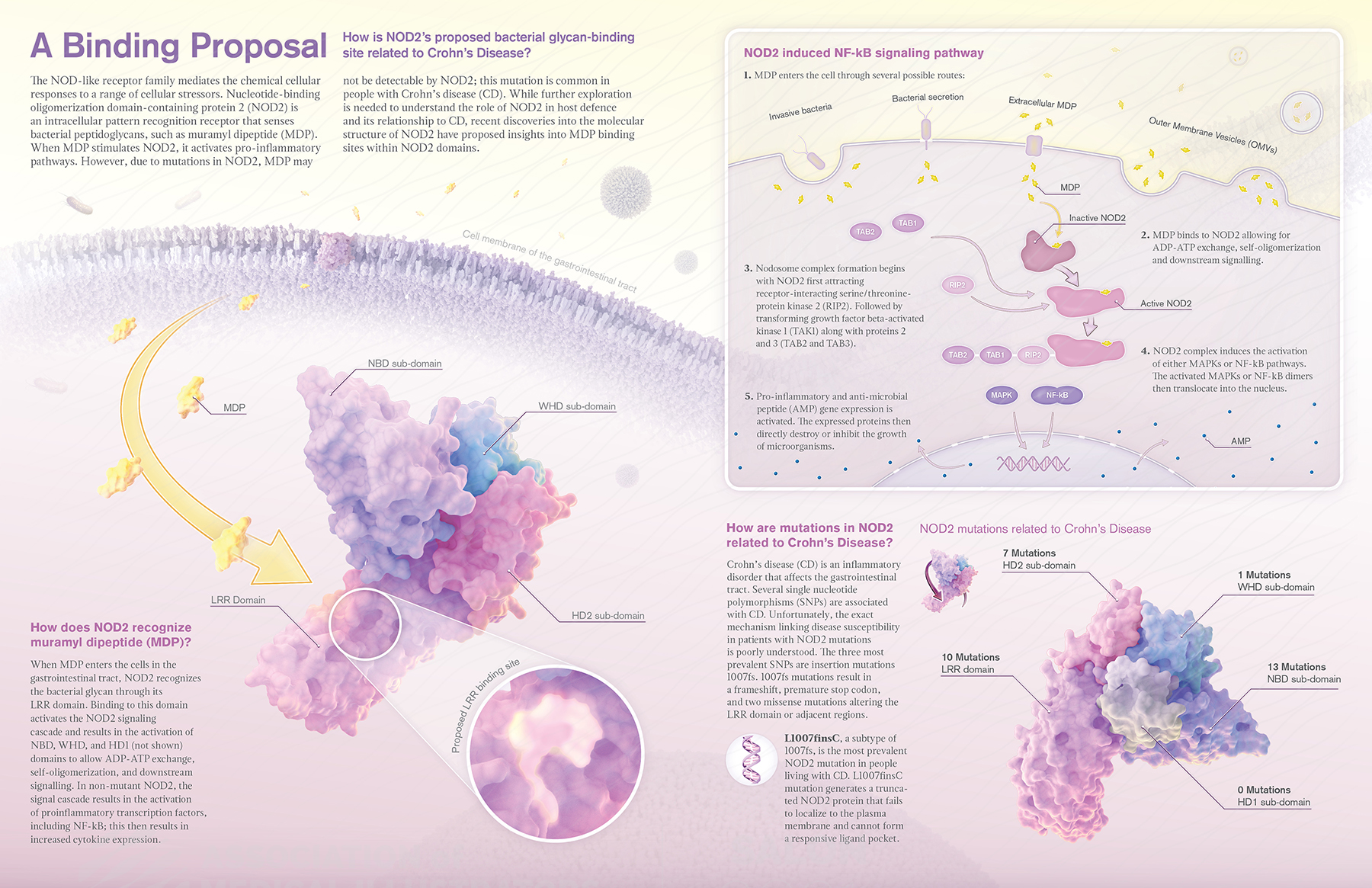

Supplement: Supplementary file 1 — Supplementary Material Details [file j_jib-2022-0016_suppl.zip › supp_material/img_src/Martin Sho 51226/a10.jpg]

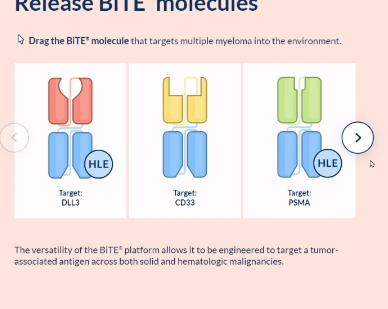

Supplement: Supplementary file 1 — Supplementary Material Details [file j_jib-2022-0016_suppl.zip › supp_material/img_src/Jason Shar 13819/a6.png]

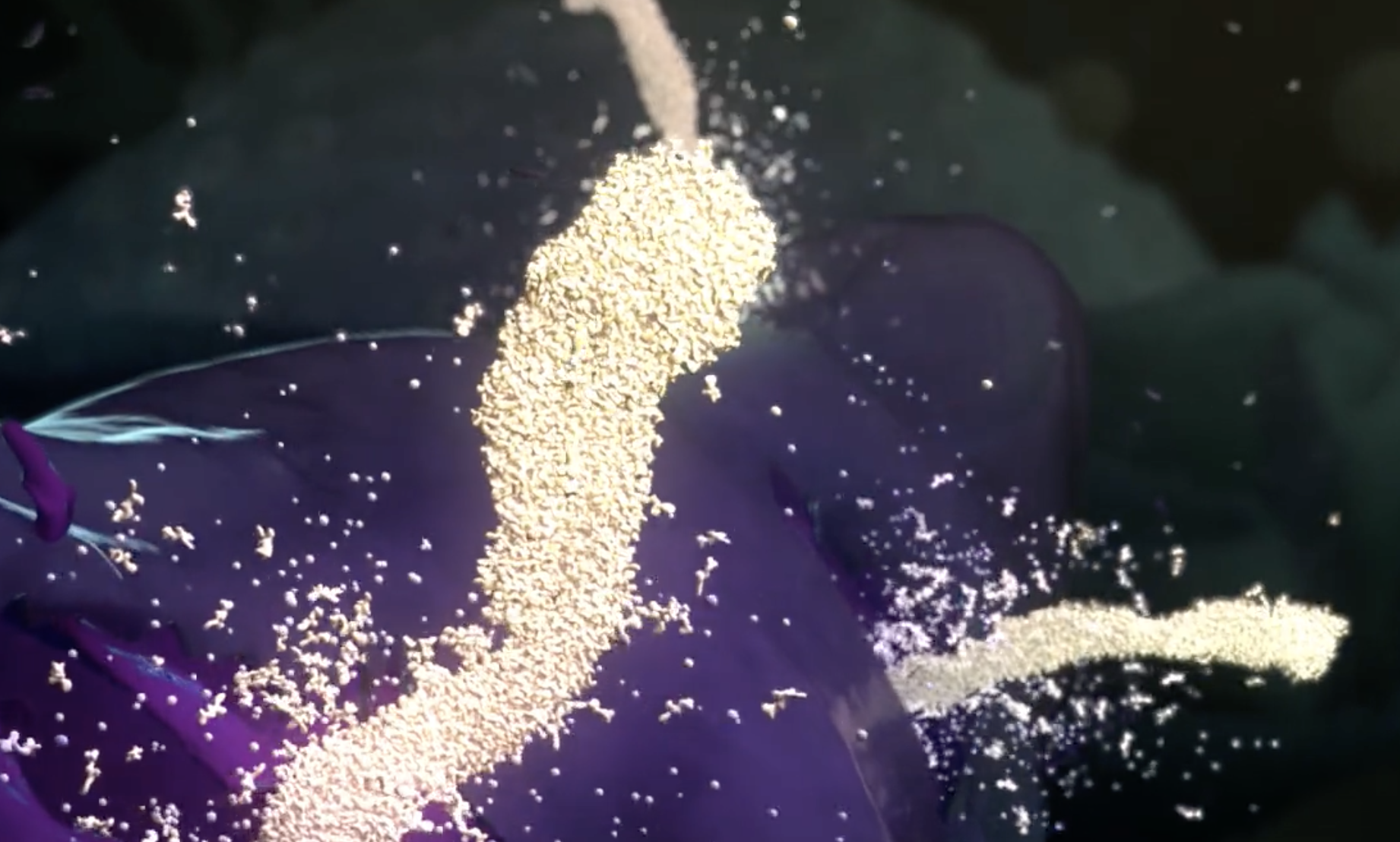

Supplement: Supplementary file 1 — Supplementary Material Details [file j_jib-2022-0016_suppl.zip › supp_material/img_src/Katie Harv be109/Screenshot_2022-02-28_at_09.57.47.png]

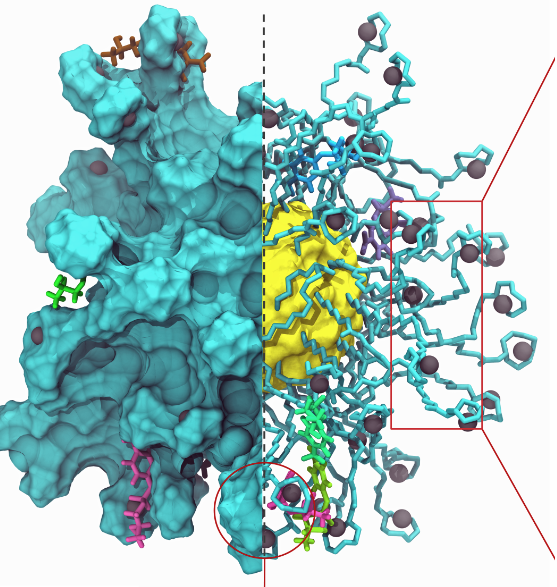

Supplement: Supplementary file 1 — Supplementary Material Details [file j_jib-2022-0016_suppl.zip › supp_material/img_src/Adam Pecin 70be1/b7.png]

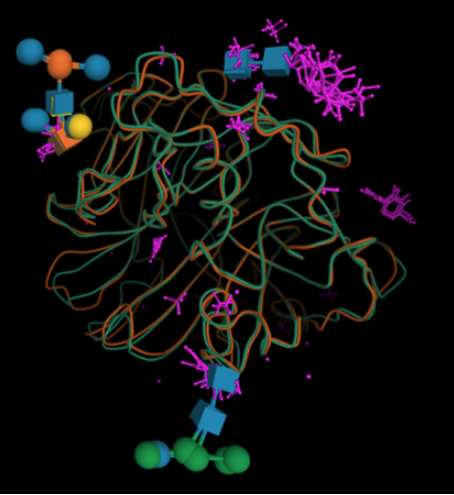

Supplement: Supplementary file 1 — Supplementary Material Details [file j_jib-2022-0016_suppl.zip › supp_material/img_src/PDB Compon fb71e/b5.png]

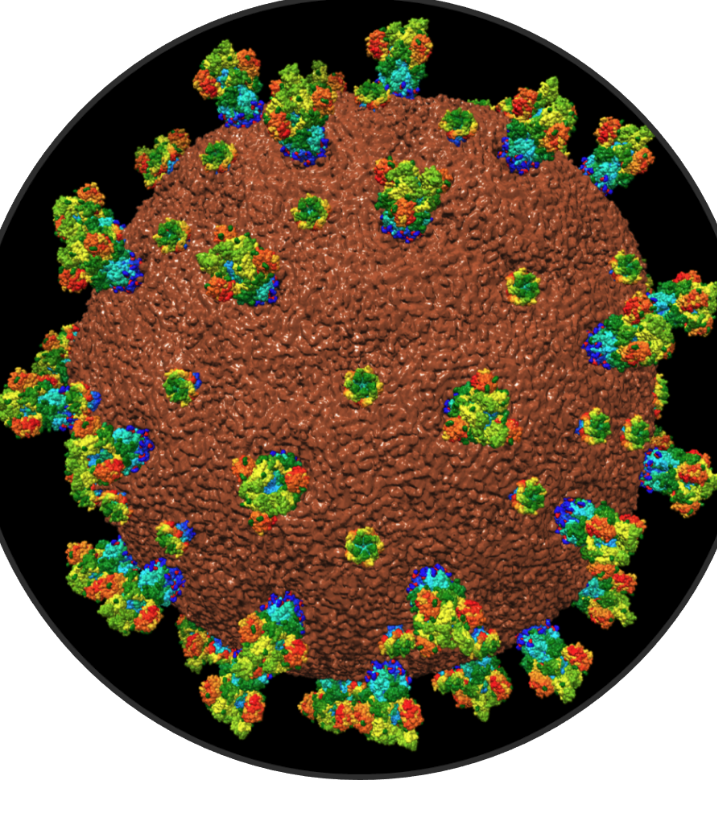

Supplement: Supplementary file 1 — Supplementary Material Details [file j_jib-2022-0016_suppl.zip › supp_material/img_src/Victor Pad 0da95/b4.png]

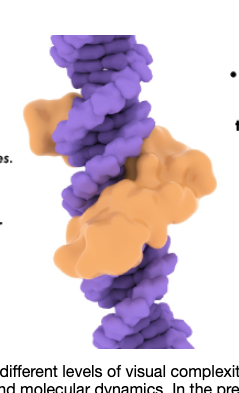

Supplement: Supplementary file 1 — Supplementary Material Details [file j_jib-2022-0016_suppl.zip › supp_material/img_src/Aygen Erge 60947/b8.png]

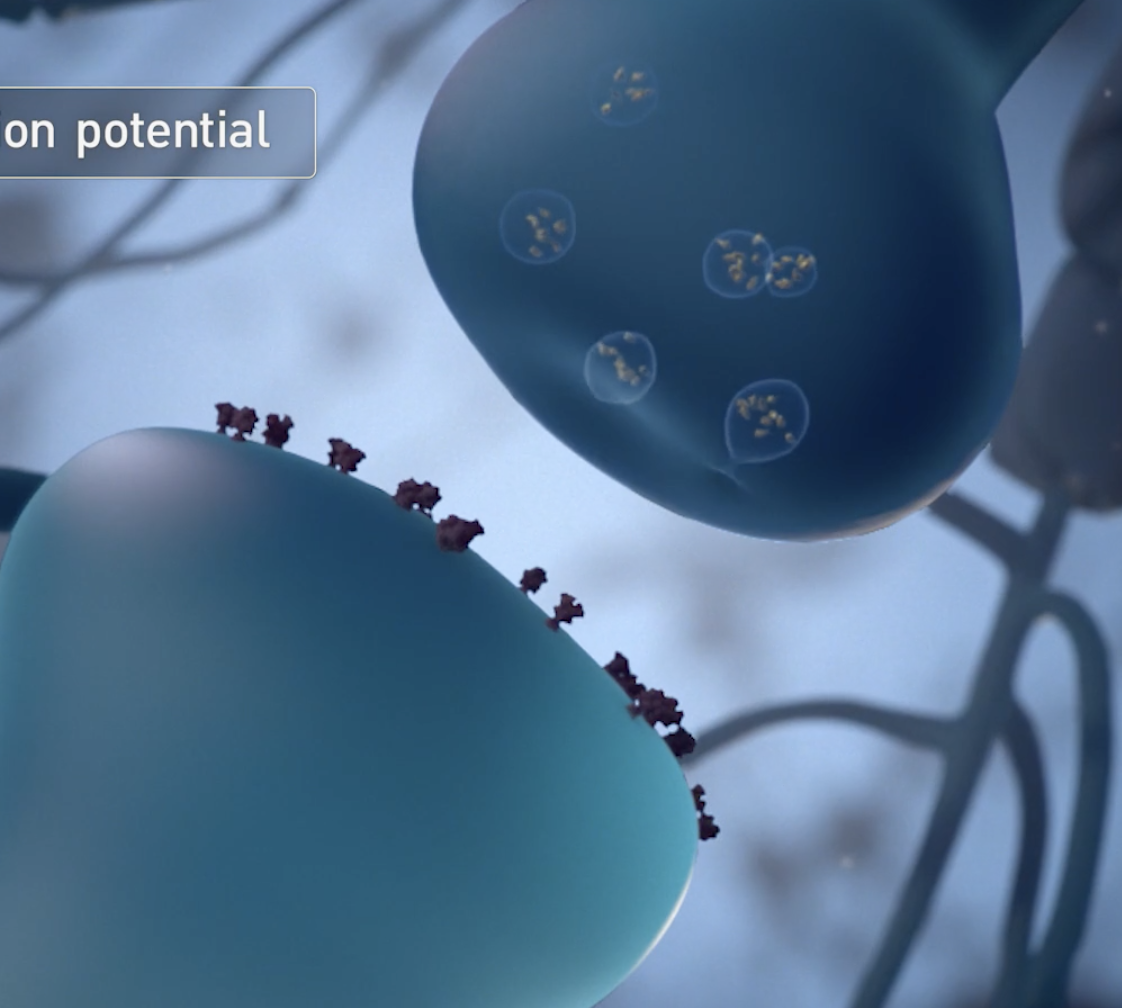

Supplement: Supplementary file 1 — Supplementary Material Details [file j_jib-2022-0016_suppl.zip › supp_material/img_src/Katherine fd7ab/Screenshot_2022-02-28_at_09.46.58.png]

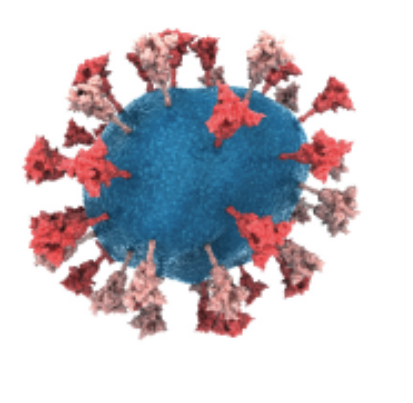

Supplement: Supplementary file 1 — Supplementary Material Details [file j_jib-2022-0016_suppl.zip › supp_material/img_src/Kristen Br 4e3ac/b6.png]

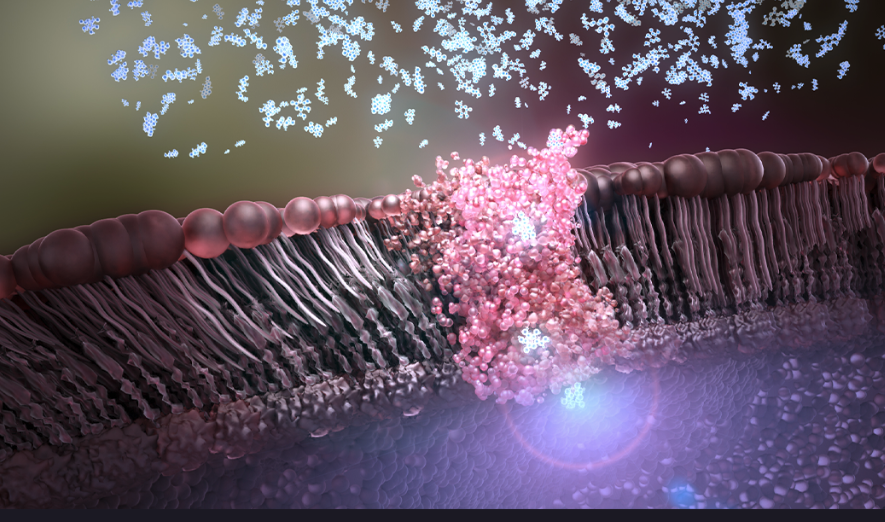

Supplement: Supplementary file 1 — Supplementary Material Details [file j_jib-2022-0016_suppl.zip › supp_material/img_src/Martina Fr f40ad/b3.png]

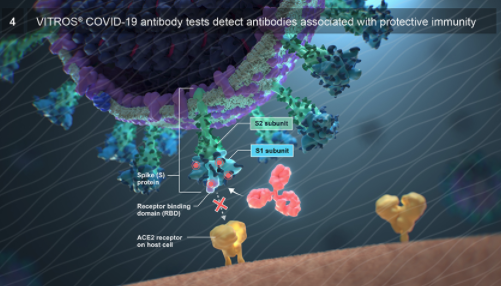

Supplement: Supplementary file 1 — Supplementary Material Details [file j_jib-2022-0016_suppl.zip › supp_material/img_src/Jason Shar a718c/a7.png]

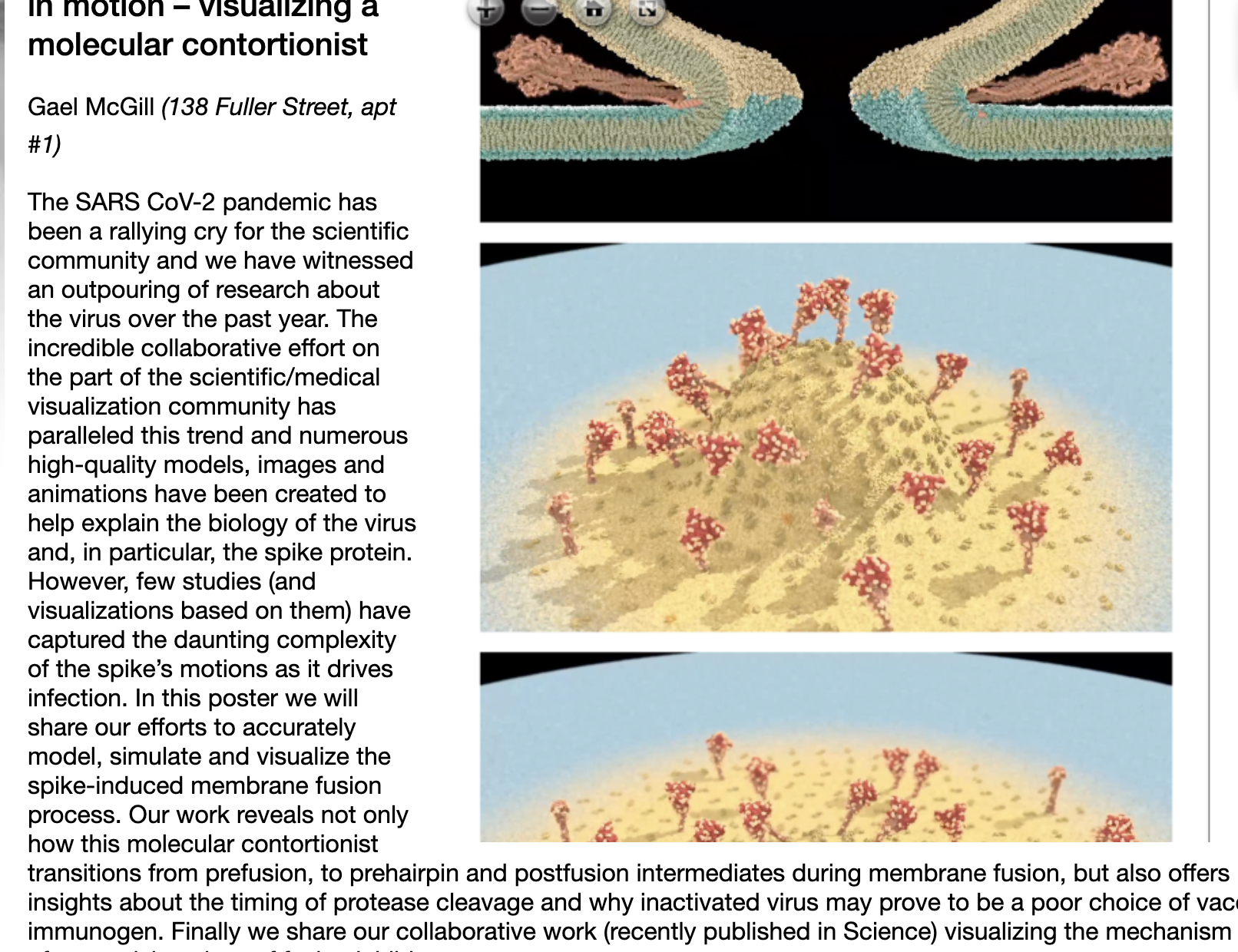

Supplement: Supplementary file 1 — Supplementary Material Details [file j_jib-2022-0016_suppl.zip › supp_material/img_src/Gael McGil 6b606/b2.png]

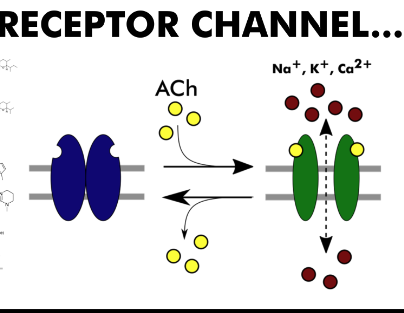

Supplement: Supplementary file 1 — Supplementary Material Details [file j_jib-2022-0016_suppl.zip › supp_material/img_src/Aliaksei C 8f2f0/b10.png]
